# NOD1 deficiency promotes an imbalance of thyroid hormones and microbiota homeostasis in mice fed high fat diet

**DOI:** 10.1038/s41598-020-69295-2

**Published:** 2020-07-23

**Authors:** Silvia González-Ramos, Marta Paz-García, Victoria Fernández-García, Kevin J. Portune, Emilio F. Acosta-Medina, Yolanda Sanz, Antonio Castrillo, Paloma Martín-Sanz, Maria Jesus Obregon, Lisardo Boscá

**Affiliations:** 1 Instituto de Investigaciones Biomédicas Alberto Sols (CSIC-UAM), Arturo Duperier 4, 28029 Madrid, Spain; 2 Centro de Investigación Biomédica en Red de Enfermedades Cardiovasculares (CIBERCV), y Hepáticas y Digestivas (CIBEREHD), ISCIII, Madrid, Spain; 3 Microbial Ecology, Nutrition and Health Research Unit, Institute of Agrochemistry and Food Technology, National Research Council (IATA-CSIC), Valencia, Spain; 4 Center for Genetic Engineering and Biotechnology, Havana, Cuba; 5 Unidad de Biomedicina. (Unidad Asociada al CSIC). Instituto de Investigaciones Biomédicas Alberto Sols (CSIC-UAM) and Universidad de Las Palmas, Gran Canaria, Spain

**Keywords:** Biochemistry, Immunology, Physiology

## Abstract

The contribution of the nucleotide-binding oligomerization domain protein NOD1 to obesity has been investigated in mice fed a high fat diet (HFD). Absence of NOD1 accelerates obesity as early as 2 weeks after feeding a HFD. The obesity was due to increases in abdominal and inguinal adipose tissues. Analysis of the resting energy expenditure showed an impaired function in NOD1-deficient animals, compatible with an alteration in thyroid hormone homeostasis. Interestingly, free thyroidal T4 increased in NOD1-deficient mice fed a HFD and the expression levels of UCP1 in brown adipose tissue were significantly lower in NOD1-deficient mice than in the wild type animals eating a HFD, thus contributing to the observed adiposity in NOD1-deficient mice. Feeding a HFD resulted in an alteration of the proinflammatory profile of these animals, with an increase in the infiltration of inflammatory cells in the liver and in the white adipose tissue, and an elevation of the circulating levels of TNF-α. In addition, alterations in the gut microbiota in NOD1-deficient mice correlate with increased vulnerability of their ecosystem to the HFD challenge and affect the immune-metabolic phenotype of obese mice. Together, the data are compatible with a protective function of NOD1 against low-grade inflammation and obesity under nutritional conditions enriched in saturated lipids. Moreover, one of the key players of this early obesity onset is a dysregulation in the metabolism and release of thyroid hormones leading to reduced energy expenditure, which represents a new role for these hormones in the metabolic actions controlled by NOD1.

## Introduction

The nucleotide-binding oligomerization domain (NOD) protein NOD1 belongs to the NOD-like receptor (NLR) family of pathogen recognition receptors. NOD1 is a cytoplasmic protein that recognizes conserved fragments found in the cell wall of many types of Gram-negative or Gram-positive bacteria^1^. NOD1 resides as an inactive monomer in the cytoplasm and, upon ligand recognition, a conformational change occurs favoring oligomerization and recruitment of the serine/threonine-protein kinase 2 (RIPK2) through homotypic CARD–CARD interactions^2,3^. RIPK2 mediates the recruitment and activation of the serine/threonine kinase TAK1, which is a prerequisite for activation of the IκB kinase complex, leading to both the canonical NF-κB and MAPK activation pathways^4^. The bacterial determinants involved in NOD1 activation have not been yet fully elucidated, but several studies have demonstrated that the minimal motif γ-d-glutamyl-meso-diaminopimelic acid (iE-DAP), present in most bacterial peptidoglycans, is sufficient to fully activate NOD1^1,2^. Although NLRs and TLRs are thought to play partial redundant roles, their expression pattern shows certain specificities that can be evidenced in genetically targeted animal models challenged with different pathogens^1,5^. NOD1 is widely expressed by a variety of cell types, but is significantly represented in epithelia, stromal and endothelial cells, adipocytes and in the thyroid^6,7^, and to a lesser extent in resting myeloid cells^3,4,8–10^. Activation of NOD1 in intestinal epithelial cells triggers the production of chemokines and the recruitment of acute inflammatory cells in vivo, playing a role in the immune response against pathogenic microbes^4,11,12^. Indeed, NOD1 KO mice are more susceptible to multiple bacterial pathogens suggesting a specific role for this receptor in the innate defense mechanisms of the intestinal epithelium against acute infections^13–16^. Moreover, NOD1 also exerts a protective role against chronic intestinal inflammatory processes, like colorectal cancer as deduced from NOD1 or RIPK2 deficient mice challenged with different colonic tumor inducers^13,15–17^, proving its role in the maintenance of intestinal homeostasis.

The innate immune system is strongly associated with obesity, insulin resistance, metabolic syndrome and diabetes^18^. Recent studies suggest that much of this interplay is due to NLR activation, in particular NOD1^18–20^. Moreover, insulin resistance has been associated with both NOD1 and NOD2 activities by eliciting pro-inflammatory signaling pathways in adipocyte cell models and in in vivo studies on human adipose tissue^20–23^. Indeed, it appears that several fatty acids can moderately activate these NLRs leading to a chronic low-grade inflammatory tone^15,24^. Several studies suggest that the intestinal barrier becomes compromised during HFD feeding, resulting in translocation of gut bacterial components into systemic circulation^18^. Therefore, intestinal epithelial NOD1 may play a protective role in strengthening the gut barrier and restricting this bacterial influx in obesity. Accordingly, mice deficient in NOD1 present an exacerbated systemic inflammation under HFD, in addition to alterations in the broad metabolic homeostasis of the animals^2,20,24,25^. In this work, we have examined the impact of a HFD in NOD1 deficient mice compared to wild-type (WT) mice during the early onset of diet-induced obesity. Our data show that NOD1 deficient mice fed a HFD for six weeks exhibit an exacerbated increase in lipogenesis in the iWAT and eWAT, but this occurs in the absence of an evident insulin resistance phenotype. In addition to this, important alterations in thyroid hormone dynamics were observed when comparing WT *vs*. NOD1 KO mice fed a HFD, which probably are involved in the progression to further metabolic dysfunctions. The NOD1 deletion also influences the composition of the gut microbiota and the HFD-induced alterations associated with a worse metabolic phenotype.

## Materials and methods

### Materials

Common reagents were from Sigma-Aldrich-Merck (St Louis, MO, USA) or Roche (Darmstadt, DE). Murine or human cytokines were obtained from PeproTech (London, UK). Antibodies were from Ambion (Austin, TX, USA), Abcam (Cambridge, UK) or Cell Signaling (Danvers, MA, USA). Reagents for electrophoresis were from Bio-Rad (Hercules, CA, USA). Tissue culture dishes were from Falcon (Lincoln Park, NJ, USA), and serum and culture media were from Invitrogen (Life Technologies/Thermo-Fisher, Madrid, ES).

### Animal care and tissue preparation

Wild type (WT) and homozygous *Nod1*^−/−^ (NOD1 KO) mice (both in the mixed C57BL/6 and 129/C57BL/6 background), have been previously described^7,26,27^. Male NOD1 WT or KO were housed under 12 h light/dark cycle and food and water was provided ad libitum (chow diet: CHD; 2.05% fat, mainly oleic and linoleic acids; SAFE-PANLAB, ES) for 12 weeks. Animals were then maintained in CHD or switched to a high-fat diet (HFD; 10.2% hydrogenated coconut fat and 0.75% cholesterol; Ssniff, Soest, DE) which was provided ad libitum for six weeks. Treatment with the NOD1 activator iE-DAP was achieved as previously described^7^. Animals were cared for according to the protocol approved by the Ethical Committee of CISC, and in accordance to directive 2010/63/EU of the European Parliament.

### Evaluation of animal metabolic profile

Animals under analysis were maintained individually in Phenomaster Metabolic Cages (TSE Systems International Group) from day 33 to day 38 and the evaluation was done during day 35–38. Spontaneous physical movement, drink and food intake, respiratory exchange (O_2_ and CO_2_), weight and heat production were monitored as previously described^28^.

### Evaluation of the glucose tolerance test

Mice underwent a glucose tolerance test (GTT) at the beginning of the HFD and at week 6 of treatment. Animals were fasted overnight and injected with 2 g of glucose per kg body weight. Blood glucose was measured with an Accu-Check glucometer (Roche) from the tail vein at 0, 15, 30, 60 and 120 min. Plasma insulin levels were determined using an ELISA kit (Mercodia, Uppsala, SE) and in parallel to the glucose determination (15 min after glucose administration).

### Assay of total bile acids (BA)

A fluorimetric assay (Sigma-Aldrich) was used to determine total bile acids in serum and in liver extracts. The assay is based on the use of 3-hydroxysteroid dehydrogenase that reacts with twelve BA, reducing NAD to NADH. Appropriate internal controls were used to ensure the linearity and specificity of the reaction.

### ^1^H NMR analysis of liver lipids

HRMAS analysis was run at 4 °C in an 11.7 T Bruker Avance spectrometer at 500.113 MHz, and using 5 kHz spinning rate, following a previously described protocol for lipids^29,30^. The ex vivo spectra were quantified by using MestReC software (Mestrelab Research, Santiago de Compostela, ES). Validation of ^1^H MR spectra were obtained from different FA standards and data were generated using heuristic analysis. Results were expressed as: SFA: saturated fatty acids (FFAA); UFA: Unsaturated FFAA; MUFA: monounsaturated FFAA; PUFA: polyunsaturated FFAA; MCL: medium chain length FFAA; NDB: number of double bonds in FFAA; PU/UFA, polyunsaturated/unsaturated fatty acids; saturation index (Sat. Index), as the ratio between stearic and oleic acids, reflecting membrane rigidity.

### Quantification of myeloid cell populations

Animals were fed as indicated and the presence of myeloid cells in the eWAT and liver at 24 h was assessed in a FACSCanto II flow cytometer as previously described^7^. The adipose tissue (eWAT) was mechanically digested using an enzymatic solution (type II collagenase, CaCl_2_ plus BSA) for 20 min at 37 °C in a rotational shaker (200 rpm). Leukocyte subsets were defined using FlowJo software: neutrophils (CD45^+^CD115^−^Ly6G^+^), inflammatory monocytes (CD45^+^CD115^+^) and tissue macrophages (CD45^+^CD115^−^F4/80^+^)-population^31^.

### Preparation of total protein cell extracts

Tissues were homogenized as previously described in the presence of 1 mM β-mercaptoethanol, detergent (0.5% CHAPS) and protease and phosphatase inhibitor cocktail from Sigma. Extracts were centrifuged and protein content was quantified^7^.

### RNA isolation and analysis

RNA was extracted in TRIzol and processed in a Real-time PCR equipment as previously described^7,32^. Samples were analyzed in duplicate *vs.* 36B4. *Dio2*,* Ucp1* and *Dio1* were assayed using specific Taqman probes (Mm01244861m1 for *Ucp1,* Mn00515664m1 for *Dio2*, Mn00839358_m1 for *Dio1* (Gene expression assays, Applied Biosystems, Foster City, CA). Results were normalized to cyclophilin labelled with VIC (*Ppia*, Mm02342429g1, Applied Biosystems) in the same well. The fold-change in mRNA expression was calculated by the 2^−ΔΔCt^ method.

### Protein analysis by Western blot

Protein cell extracts were boiled in 250 mM Tris–HCl, pH 6.8; 2% SDS, 10% glycerol and 2% β-mercaptoethanol, and equal amounts of protein were separated by SDS-PAGE electrophoresis in 8–10% gels and visualized by ECL chemiluminescence as described^7,32^. The antibodies used were from Abcam or Cell Signaling.

### Thyroid hormones determinations

The concentrations of T3 and T4 were measured after extraction and purification from plasma, liver, brown adipose tissue (BAT) and thyroids using specific radioimmunoassays. For hormone measurement in thyroids, tissues were homogenized in ethanol, and thyroidal free (F) T4 and T3 were determined in the dried extracts as described^33^.

### Deiodinase activities

#### D2 in BAT and D1 in liver

^125^I-T4 and ^125^I-rT3 were synthetized and purified to reduce iodide to less than 1% of the total ^125^I. D2 activity was assayed as previously described^34,35^. D1 activity was determined as previously described^36^.

### Histological analysis and immunostaining

Mouse liver and eWAT tissues were fixed in 4% paraformaldehyde in 0.1 M PBS and embedded in paraffin. 5 μm sections of tissue were deparaffinized sections were stained with hematoxylin/eosin (HE) or Oil-Red O using standard procedures.

### Microbiota analysis

#### Analysis of gut microbiota

Fecal samples from individual mice from each experimental group (n = 10 for control, n = 8 for NOD1 KO) were collected after 12 weeks on a CHD and then at 6 weeks after the switch to the HFD and stored in 2 ml cryovials. Samples were immediately frozen in liquid nitrogen and maintained at − 80 °C. DNA extraction was done using Fast DNA Stool Mini Kit (Qiagen) according to the manufacturer’s instructions with several modifications: Fecal samples (*ca*. 220 mg) were homogenized with glass beads in the presence of 1 ml of Inhibitex buffer (Qiagen) using a beadbeater for 2 successive rounds for 1 min and then heated to 95 °C for 10 min. DNA was PCR-amplified in triplicate using primers (S-D-Bact-0341-b-S-17/S-D-Bact-0785-a-A-21) that target the V4–V5 variable regions of the 16S rRNA gene, following a previous protocol^37^. Samples were tagged with barcodes to allow multiplexing during the sequencing process. Sample amplicons were combined and purified using the Illustra GFX PCR DNA and Gel Band Purification Kit (GE Healthcare) according to the manufacturer’s instructions and combined in equimolar concentrations before carrying out sequencing on a MiSeq instrument (Illumina). All raw sequence data has been submitted to ENA-EMBL Accession # PRJEB33681.

Bioinformatic processing of data was carried out using the software QIIME1 (version 1.9.1)^38^, Mothur^39^, and UPARSE^40^. Briefly, using QIIME1, paired-end forward and reverse Illumina reads were joined into contigs (join_paired_ends.py), barcodes were extracted (extract_barcodes.py), and reads were demultiplexed (split_libraries_fastq.py). Sequences smaller than 325 bp or containing homopolymers greater than 7 were removed using the screen.seqs() command from the software program Mothur. Primers were then removed using trim.seqs() from Mothur. Using UPARSE, chimeras were removed and reads were clustered at 97% identity into OTUs using default settings. An OTU abundance table was generated within the UPARSE pipeline by mapping reads to representative sequences for each OTU. Using QIIME, a biom file was created from the OTU table and reads were rarefied (single_rarefaction.py) and singletons were removed (filter_otus_from_otu_table.py). A phylogenetic tree was constructed from representative sequences for each OTU, and aligned (align_seqs.py) using PYNAST^41^ and filtered (filter_alignment.py) using default settings. Alpha diversity metrics (Shannon’s, Chao1, observed OTUs) were calculated (alpha_diversity.py). Beta diversity analysis was conducted using generalized UniFrac (GUniFrac)^42^. Taxonomy was assigned to representative OTUs (summarize_taxa.py) using UCLUST against the Greengenes database (version 13.8).

#### Statistical analyses of microbiota

The biomarker discovery method LDA Effect Size (LEfSe^43^) was employed to compare the effect of genotype (WT vs. NOD1 KO groups) under a CHD and HFD diet, as well as examine the effects of a HFD after 6 weeks. Comparisons of beta diversity between groups using generalized UniFrac were performed by a PERMANOVA with adonis within the GUniFrac package in R.

### Statistical analysis

After normality, the statistical analysis was calculated by D’Agostino-Pearson omnibus test, either a nonparametric test (Mann–Whitney U test), or a normality test (unpaired Student’s t test with Welch’s correction, ordinary 1-way ANOVA) was used as appropriate. Removal of outliers was assessed by the ROUT method. Results were considered statistically significant at *P* < *0.05*.

## Results

### Loss of NOD1 accelerates diet-induced obesity

**Figure 1:**
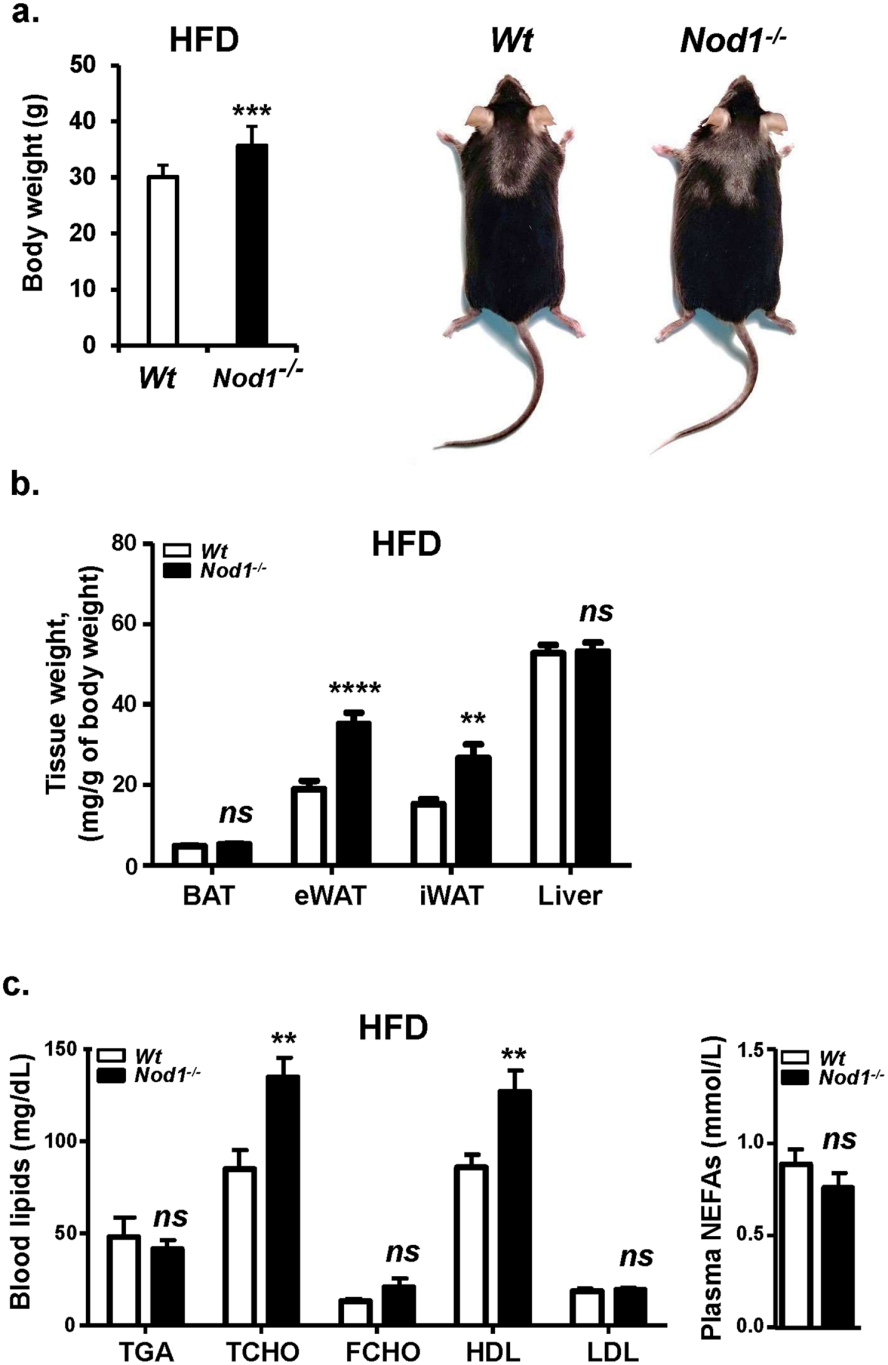
NOD1 deficient mice exhibit enhanced body weight and obesity after feeding high-fat diet (HFD). 12-weeks old WT (n = 27) and NOD1 KO mice (n = 26) maintained in chow diet (CHD) were fed for 6 additional weeks with CHD or HFD. (**a**) Body weight and representative images of animals. (**b**) Adipose tissues (brown adipose tissue, BAT; epididymal adipose tissue, eWAT; inguinal adipose tissue, iWAT) and liver weight under 6-weeks HFD conditions**. **(**c**) Serum lipid profile and non-esterified fatty acids content from these animals (n = 7, for each group). Results show the mean ± SEM of the indicated samples. **P < 0.01; ***P < 0.005; ****P < 0.001 vs. the corresponding condition in WT mice. *ns* not statistically significant.

**Figure 2:**
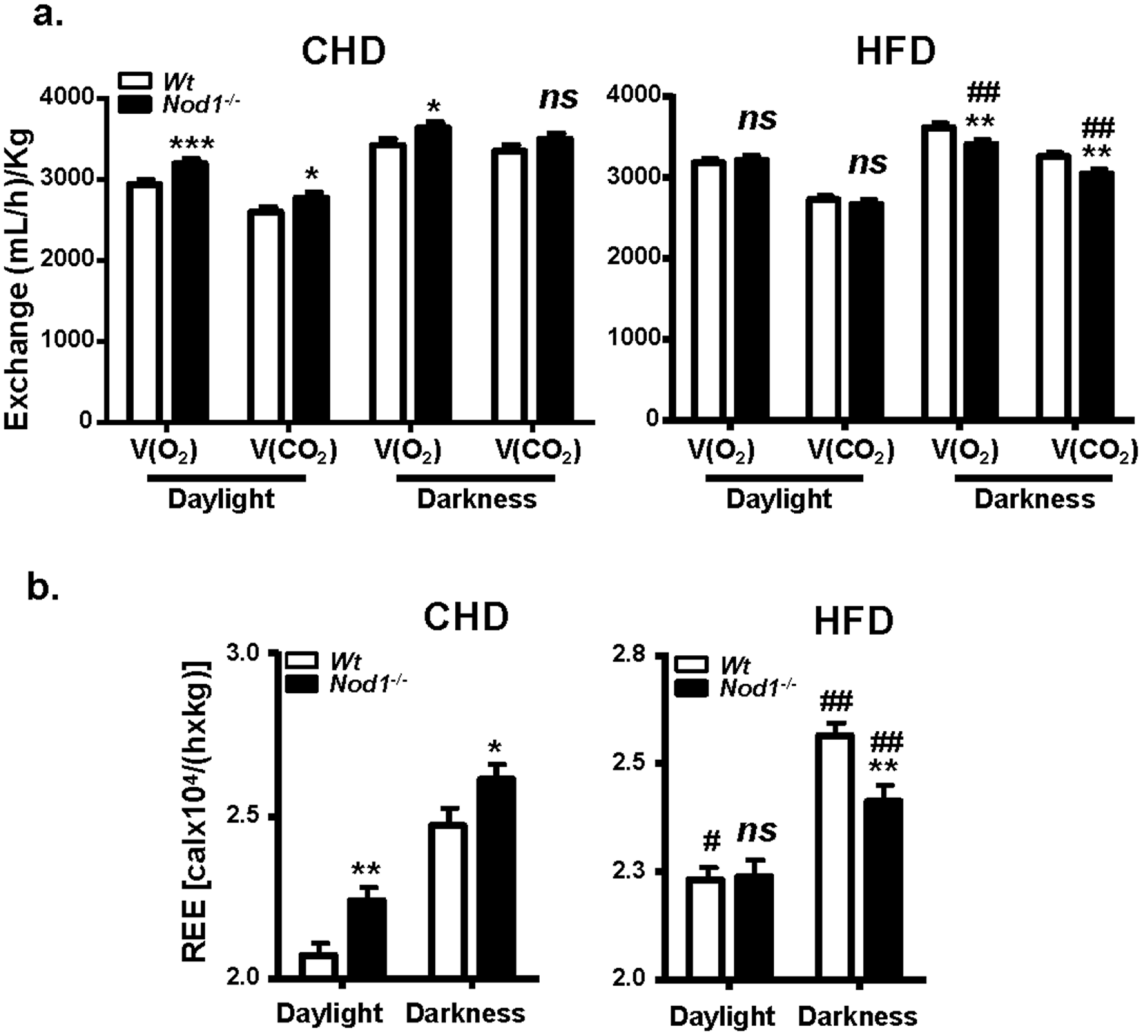
Evaluation of animal metabolic profiles. WT (n = 12) and NOD1 KO (n = 12) animals were fed standard chow diet (CHD) or high-fat diet (HFD) for 6 weeks. The O_2_ and CO_2_ exchange (**a**), the Resting Energy Expenditure (REE) (**b**) were registered as described in the “Materials and methods” section, during the daylight and darkness periods (12 h). Results show the mean ± SEM of 6 animals per nutritional condition and genetic background. *P < 0.05; **P < 0.01; ***P < 0.005 vs. the corresponding condition in WT mice; ^*#*^*P* < 0.05, ^*##*^*P* < 0.01 vs. the corresponding condition under HFD. *ns* not statistically significant.

**Figure 3:**
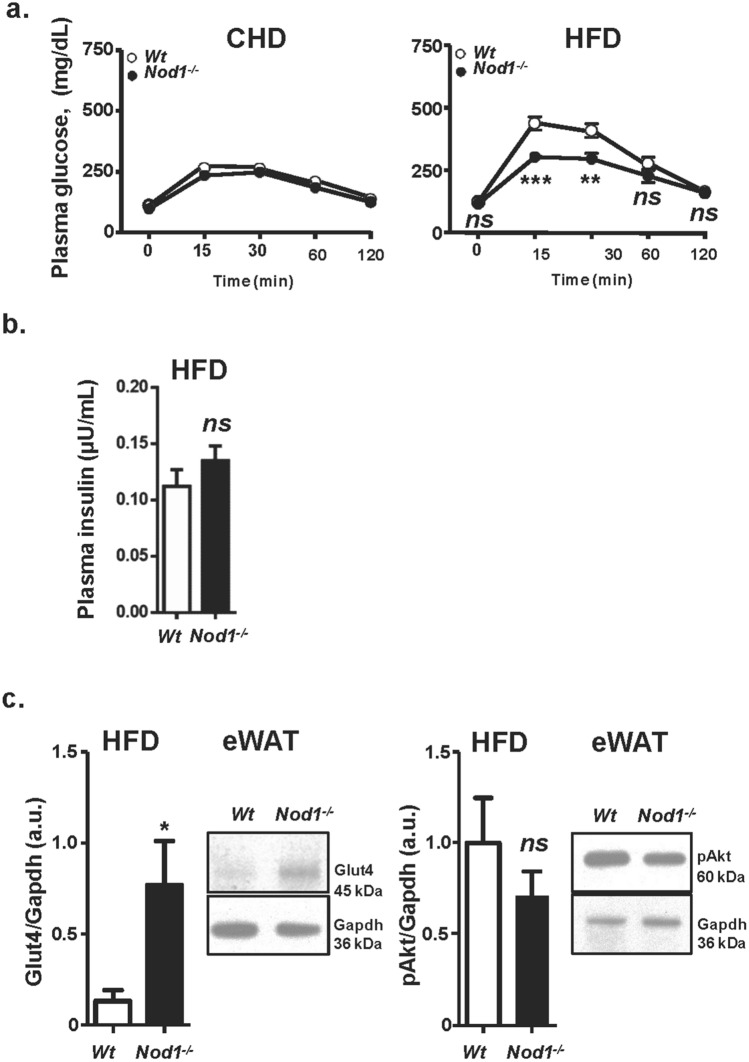
NOD1 KO mice fed HFD exhibit improved glucose tolerance. WT and NOD1 KO mice were fed CHD or HFD for 6 weeks and, at the end of the period were starved and submitted to a glucose tolerance test. Plasma glucose level after i.p. administration of 2 g of glucose per kg body weight (**a**). Plasma insulin levels from these animals were determined 15 min after glucose administration (**b**). Immunoblot levels of Glut4 and pAkt in eWAT (**c**). Results show the mean ± SEM of 6 animals per nutritional condition and genetic background. *P < 0.05; **P < 0.01; ***P < 0.005 vs. the corresponding condition in WT mice. *ns* not statistically significant. Immunoblot corresponds to a representative assay out of four.

WT and NOD1-deficient mice fed standard CHD did not show differences in body weight across the lifetime of the animals (not shown). However, after 6 weeks on HFD, statistically significant differences in body weight were observed, mainly due to fat accumulation in the eWAT, iWAT and BAT (Supplementary Fig. S1a, Fig. 1a,b), but not in other organs (e.g., liver; Fig. 1b). The increase in body weight of NOD1-deficient mice fed HFD was statistically significant after two weeks of treatment, having both groups the same tibiae size (Supplementary Fig. S1a,b). Regarding blood lipids, HFD-fed mice exhibited an increase in total cholesterol (TCHO) due to an elevation in the HDL fraction, but no differences were observed in other lipids assayed, including triglycerides (TGA) and non-esterified fatty acids (NEFA; Fig. 1c). When animals were analyzed in metabolic cages, NOD1-deficiency in mice fed CHD resulted in higher respiratory activity. However, a depressed respiration during the darkness period was observed in NOD1-deficient mice *vs*. the WT counterparts when fed a HFD (Fig. 2a). These metabolic differences included a substantial decrease in Resting Energy Expenditure (REE) during darkness and in lesser motion in HFD NOD1 KO animals, a situation opposite to that observed under CHD (Fig. 2b, and Supplementary Fig. S2), suggesting a role for thyroid hormones (a key player in REE) in this differential response^44^. However, no significant differences in body temperature (not shown), and food and beverage intake were observed between WT and NOD1 KO mice regardless the dietary regimen (Supplementary Fig. S2). To further investigate the mechanisms associated with the observed body weight gain, the glucose tolerance test was evaluated. No differences in the time-dependent glucose drop were observed in animals under CHD; however, NOD1 KO mice fed HFD exhibited an unexpected improvement in the glucose tolerance test that was not due to differences in plasma insulin levels (Fig. 3a,b). These data are compatible with increased GLUT-4 expression from eWAT in NOD1 KO mice, suggesting improved glucose homeostasis (Fig. 3c). Nevertheless, no significant differences in phospho-AKT were observed under these conditions (Fig. 3c).

### NOD1 deficiency increases white adipocyte lipid content and promotes liver steatosis in HFD-fed mice

**Figure 4:**
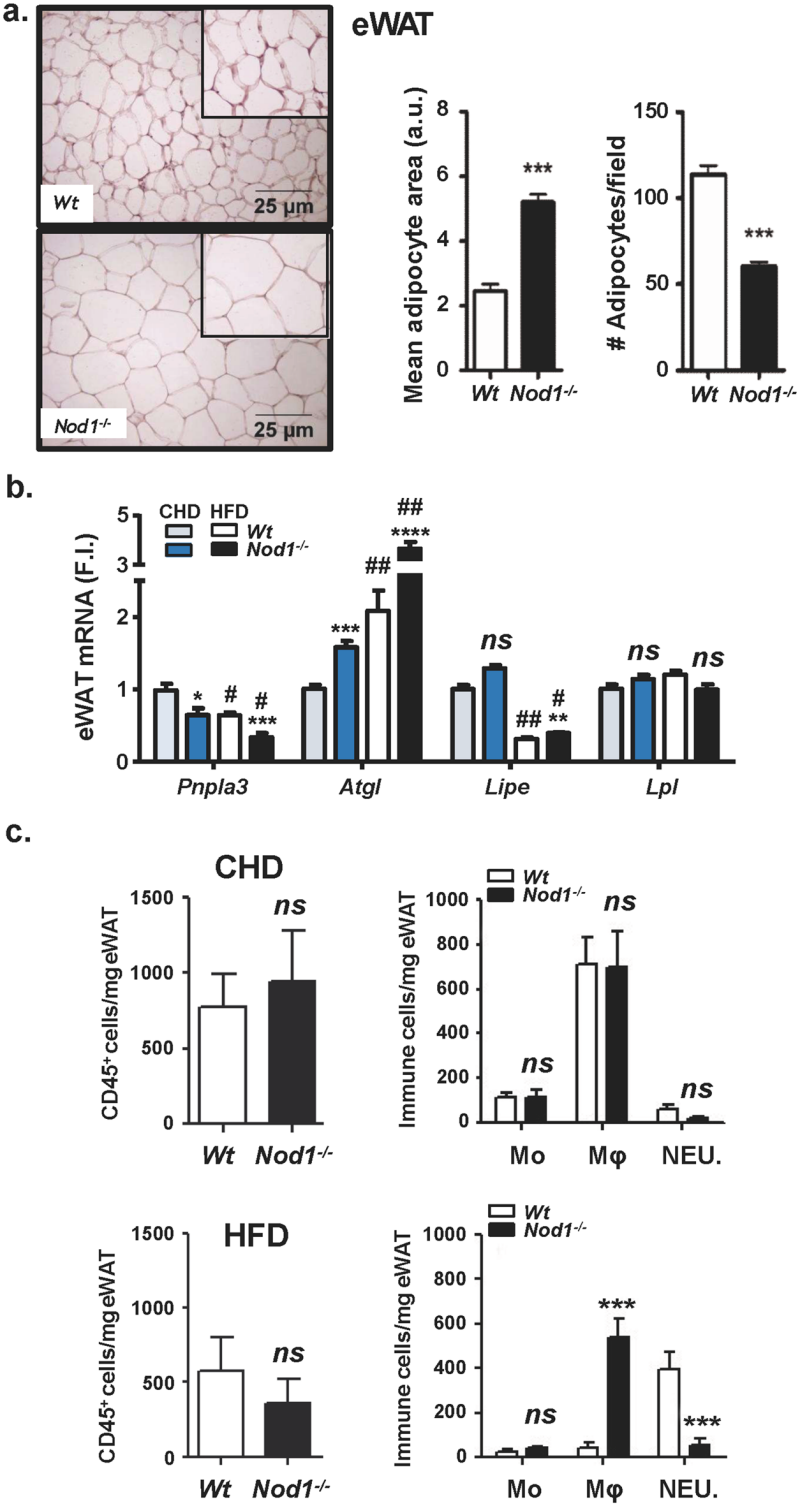

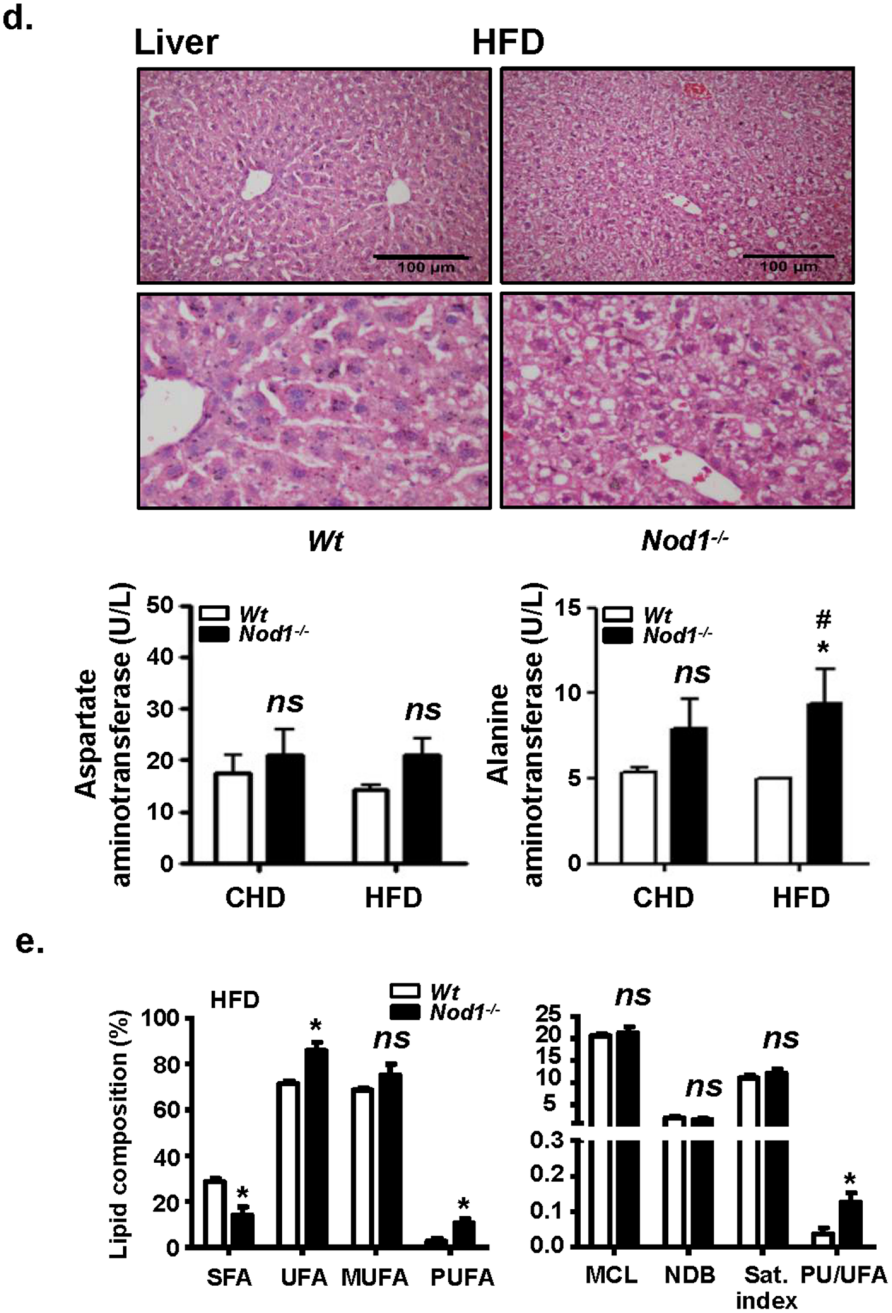
NOD1 KO mice fed HFD exhibit a significant increase in eWAT size, infiltration of immune cells and hepatic steatosis. Immunohistochemical analysis of eWAT from WT and NOD1 KO mice fed HFD for 6 weeks, and evaluation of adipocyte area and cell density (**a**). qPCR analysis of eWAT lipases from mice fed CHD or HFD for 6 weeks (**b**). Infiltration of immune cells in eWAT (**c**). Accumulation of lipids in liver and serum transaminase levels from WT and NOD1 KO mice fed HFD (**d**). Hepatic lipid composition from HFD fed animals determined by ^1^H magnetic resonance spectrum (^1^H MRS-HRMAS) (**e**). Results show the mean ± SEM of 4 (**b**, **c**) and 6 (**a**, **d**, **e**) animals per nutritional condition and genetic background. *P < 0.05; **P < 0.01; ***P < 0.005; ****P < 0.001 vs. the corresponding condition in WT mice. ^*#*^*P* < 0.05, ^*##*^*P* < 0.01, ^*###*^*P* < 0.005 vs. the corresponding genetic condition under CHD. *Ns* not statistically significant. Results were expressed as: SFA: saturated fatty acids (FFAA); UFA: Unsaturated FFAA; MUFA: monounsaturated FFAA; PUFA: polyunsaturated FFAA; MCL: medium chain length FFAA; NDB: number of double bonds in FFAA; PU/UFA, polyunsaturated/unsaturated fatty acids; saturation index (Sat. Index), as the ratio between stearic and oleic acids.

Since eWAT seems to be one of the tissues responsible for the increased body weight in NOD1 KO mice fed HFD, a histological analysis of this tissue was performed. In agreement with the body weight increase of NOD1 KO mice, a very significant enlargement of adipocytes was observed, indicating obesity or at least overweight (Fig. 4a). The RNA levels of key adipocyte lipases and lipid metabolism genes were determined (Fig. 4b). The phospholipase *Pnpla3* (patatin-like phospholipase domain-containing protein 3; *Pnpla3*), which is upregulated by T3 and mediates the hydrolysis of triglycerides in adipocytes playing a role on lipid storage homeostasis^45,46^, was decreased in NOD1 KO adipose tissue from CHD and HFD mice, a situation compatible with hypothyroidism. The lipase *Atgl* (patatin-*like* phospholipase domain-containing protein 2) increased significantly under HFD^21,47^. Other lipases, such as *Lipe* (hormone-sensitive lipase), decreased in HFD, in agreement to their role in controlling lipolysis^47,48^, whereas *Lpl* (lipoprotein lipase) remained unchanged in all conditions assayed (Fig. 4b). Among other genes involved in lipid homeostasis, a marked increase in *Ppara*, but not in *Pparg* was evidenced in the absence of NOD1 (not shown). Because lipid accumulation is evident in eWAT from NOD1 KO animals fed HFD, it can be suggested that lipogenesis is markedly enhanced in this tissue through mechanisms not yet fully established. Since infiltrating immune cells may influence the physiology of adipocytes, eWAT resident immune cell populations were determined by flow cytometry. As Fig. 4c shows, no significant differences in the content of CD45-positive cells or in the tissue distribution of myeloid cells was observed under CHD. However, after HFD, the eWAT tissue from NOD1 KO mice exhibited a large infiltration of macrophages, but with lesser neutrophilia (Fig. 4c, lower panel), despite the larger size of the eWAT adipocytes from NOD1 KO HFD fed mice. In addition to eWAT, the liver from NOD1 KO mice under HFD presented a significant steatosis, without elevation of serum transaminases except for a modest, although statistically significant increase in alanine aminotransferase (ALT; Fig. 4d). This increased lipogenesis in the absence of NOD1 under HFD was confirmed by the fatty acid composition of livers that showed an increase of polyunsaturated fatty acids (PUFA) and a decrease of saturated fatty acids (SFA), a metabolic situation associated with the development of non-alcoholic fatty liver disease (NAFLD)^49^ (Fig. 4e). Interestingly, WT mice fed a HFD for 4 weeks followed by 2 weeks on CHD fully recovered from the lipid accumulation in liver, whereas NOD1 KO mice under the same nutritional protocol still exhibited clear signs of steatosis and lipid accumulation in the liver (Supplementary Fig. S3). Indeed, HFD significantly activates NOD1 signaling cascade in WT mice through the phosphorylation of RIP2 kinase (Supplementary Fig. S4).

### NOD1-deficiency influences bile acid metabolism, inflammation and intestinal lipids in HFD-fed mice

**Figure 5:**
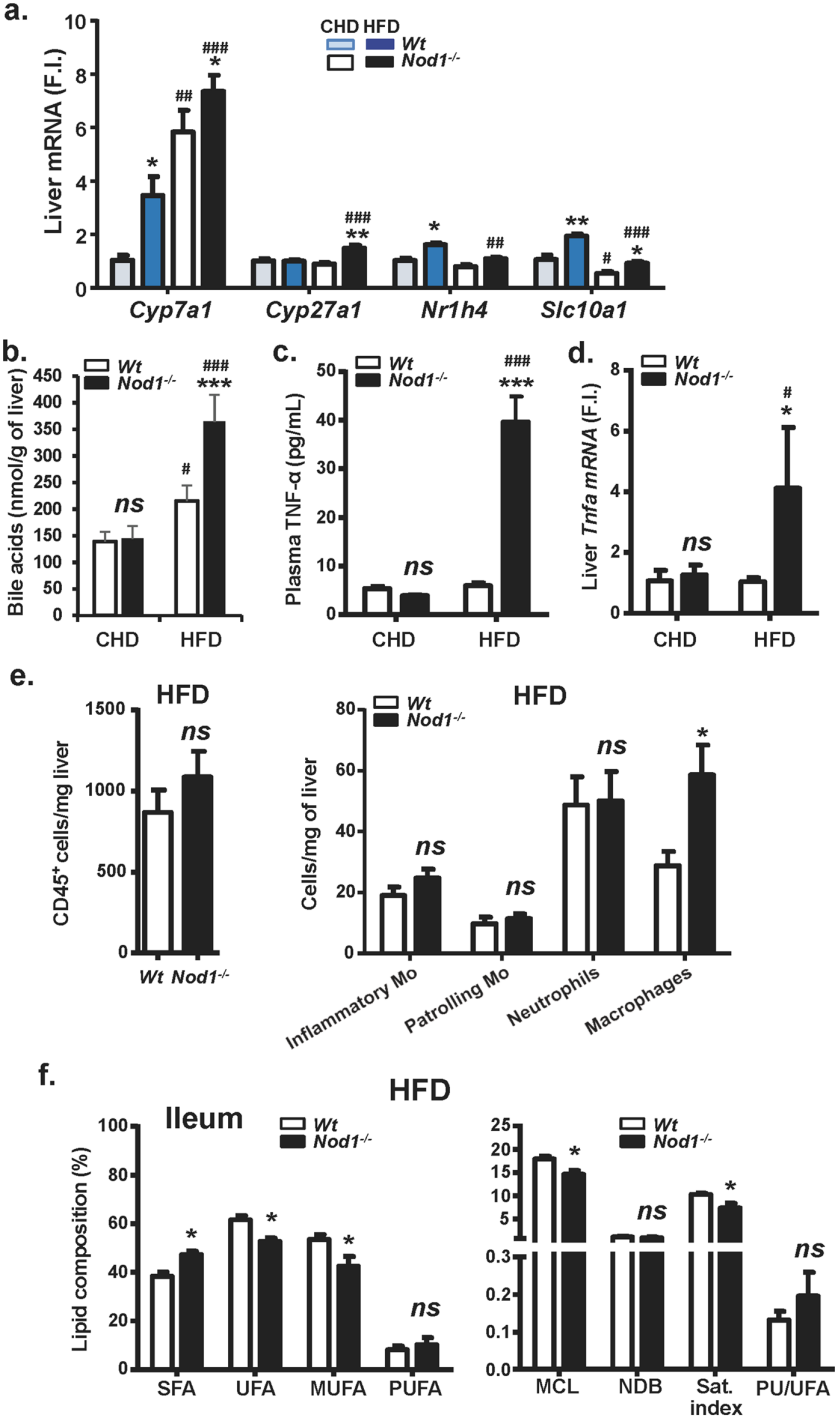
NOD1 KO mice fed HFD exhibit an enhanced liver pro-inflammatory profile. WT and NOD1 KO mice were maintained under CHD or HFD for 6 weeks. The hepatic mRNA levels of genes involved in bile acid metabolism were determined (**a**). Quantification of hepatic bile acids (**b**). The plasma levels of TNF-α (**c**) and the hepatic mRNA levels of *Tnfa* (**d**) were determined. The infiltration of immune cells in liver were determined by flowcytometry (**e**). The lipid composition of the ileum was determined by ^1^H MR spectroscopy (**f.**). Results show the mean ± SEM of 4 (**b**–**e**) and 8 (**a**, **f**) animals per nutritional condition and genetic background. *P < 0.05; **P < 0.01; ***P < 0.005 vs. the corresponding condition in WT mice. ^*#*^*P* < 0.05, ^*##*^*P* < 0.01, ^###^P < 0.005 vs. the corresponding genetic condition under CHD. *ns* not statistically significant. Results were expressed as: SFA: saturated fatty acids (FFAA); UFA: Unsaturated FFAA; MUFA: monounsaturated FFAA; PUFA: polyunsaturated FFAA; MCL: medium chain length FFAA; NDB: number of double bonds in FFAA; PU/UFA, polyunsaturated/unsaturated fatty acids; saturation index (Sat. Index), as the ratio between stearic and oleic acids.

Bile acids (BA), produced in the liver, stimulate energy expenditure through increases in D2 deiodinase in BAT. In addition to this, BA are critically important for balanced lipid metabolism and innate immunity^19,50^. Thus, we investigated the mRNA levels of relevant genes involved in cholesterol catabolism leading to BA biosynthesis (Fig. 5a, Supplementary Fig. S5). The key sterol hydrolases *Cyp7a1* (also increased in NOD1 KO CHD-fed mice) and *Cyp27a1* were significantly increased in NOD1 KO HFD fed mice, compatible with an increase in BA synthesis in NOD1 KO liver (Fig. 5b), due to the elevated levels of cholesterol under these conditions. The hepatic levels of the Na^+^/BA-cotransporter *Slc10a1* were significantly higher in NOD1 KO *vs.* WT mice in CHD regimen, suggesting that the enterohepatic circulation of BA into hepatocytes was reduced upon HFD treatment. Regarding *Nr1h4*, the farnesoid X receptor that counteracts the expression of *Cyp7a1,* avoiding excess of BA synthesis, only a minimal but statistically significant decrease was observed in NOD1 KO livers under HFD *vs*. CHD. Furthermore, in vivo NOD1 activation with iE-DAP induced *Cyp7a1* in liver as did the HFD, at the time that decreased the transcription of *Slc10a1* (Supplementary Fig. S5), suggesting the existence of alternative mechanisms independent of NOD1 and responsible for the elevation in the levels of both genes under HFD. Moreover, increased serum TNFα levels were observed in NOD1 KO mice fed HFD, probably due to the enhanced liver *Tnfa* expression measured in these animals (Fig. 5c,d). Interestingly enough, an increase, although not statistically significant, in the liver resident inflammatory monocytes was observed in these animals, that was associated with a statistically significant increase in liver macrophages (Fig. 5e). The lipid composition of intestinal mucosa from NOD1 KO mice fed HFD included a higher proportion of saturated fatty acids and a lower amount of monounsaturated fatty acids *vs*. their WT counterparts (Fig. 5f).

### Thyroid function is altered in mice lacking NOD1

**Figure 6:**
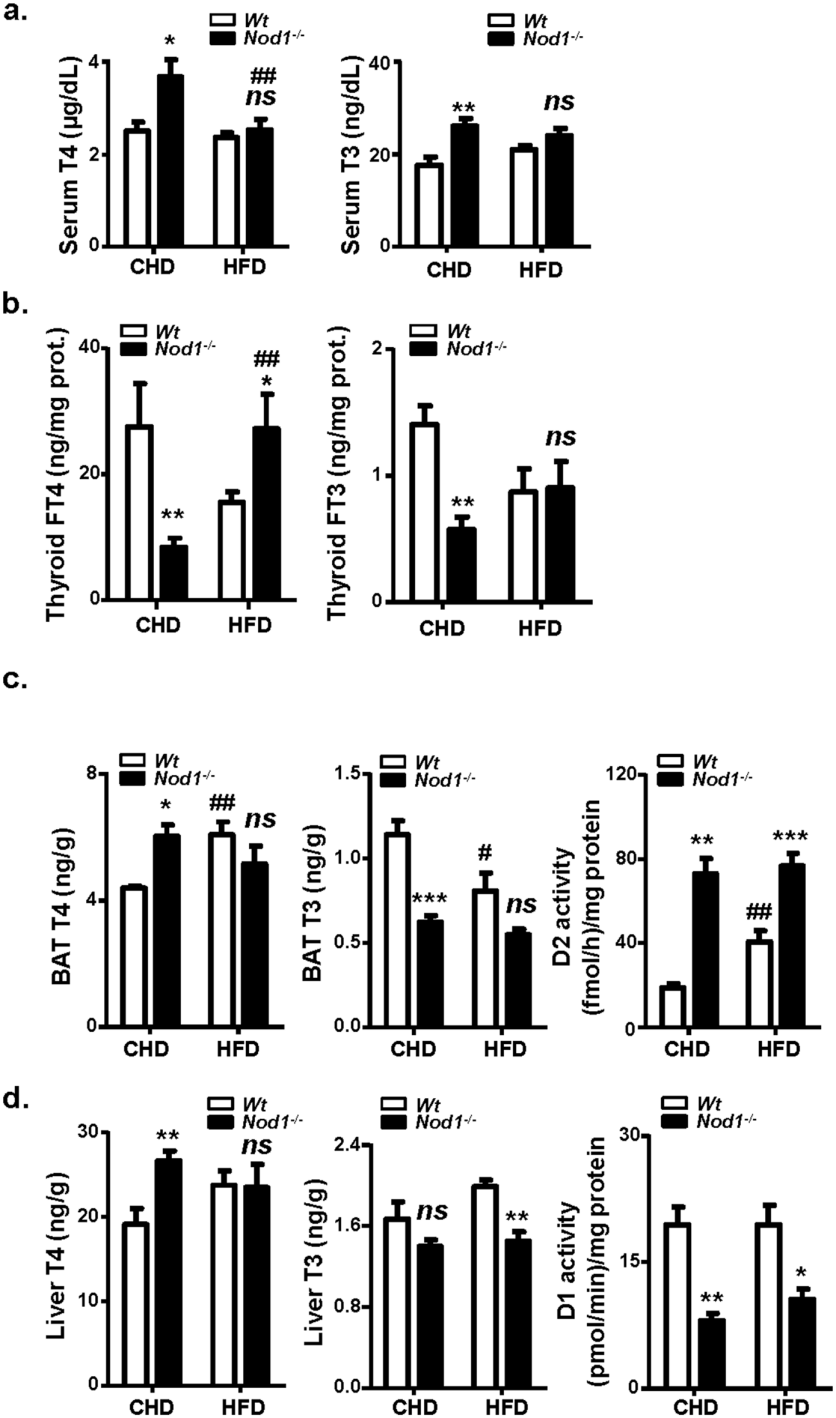
Thyroid hormones in plasma, thyroid, BAT and liver; liver D1 and BAT D2 deiodinase activities in WT and NOD1 KO mice fed CHD or HFD. Animals were maintained on CHD or fed HFD for 6 weeks. The serum (**a**) and free thyroidal concentrations (**b**) of T4 T3 were determined. The BAT T4, T3 and T4 type 2 deiodinase activity (D2 activity) were determined (**c**). The liver T4, T3 and type 1 deiodinase activity (D1 activity) were determined (**d**). Results show the mean ± S.E.M. of 9–15 animals per nutritional condition and genetic background. *P < 0.05; **P < 0.01; ***P < 0.005 vs. the corresponding condition in WT mice. ^#^*P* < 0.05; ^##^*P* < 0.01 vs. the corresponding genetic condition under CHD. *ns* not statistically significant.

Since thyroid hormones have an impact on tissue homeostasis and lipid metabolism^51–53^ as well as in energy expenditure^44^, we investigated their levels in WT and NOD1 KO mice fed CHD or HFD. The serum levels of T4 and T3 were significantly higher in NOD1 KO *vs*. WT mice under CHD, suggesting increased requirements of T4 and T3 in NOD1 KO mice. However, feeding HFD suppressed the differences between both groups (Fig. 6a). Measurement of free thyroidal T4 and T3 (thyroid FT4 and FT3 ready to be released to serum) in NOD1 KO mice fed CHD showed a strong decrease as compared to the WT, indicating damage in the secretion of T4 and T3 in NOD1 KO mice. However, in HFD, the FT4 is higher in the thyroid of NOD1 KO animals compared with WT counterparts (Fig. 6a,b). Due to the importance of BAT in regulating thermogenesis and energy balance, thyroid hormones levels were assessed in BAT and in liver. We found that T4 concentrations were increased in BAT and liver of NOD1 KO animals fed CHD in parallel to plasma T4, but feeding HFD also suppressed this T4 rise. Regarding T3 levels, absence of NOD1 resulted in equal or low T3 values than in the WT counterparts in BAT and liver, independently of the diet (Fig. 6c,d). However, deficiency in NOD1 results in statistically higher D2 activity in BAT, irrespectively of the nutritional regime of the animals (Fig. 6c) and nine- to ten-fold increases in *Dio2* mRNA (Supplementary Fig. S6a), suggesting high activation of BAT machinery to maintain T3 levels in BAT. Of note, that this occurs with a normal or high BAT T4 (low T4 is the main activator of D2 activity), that excludes a role for T4 in the D2 activity and *Dio2* mRNA increases, pointing to other activators for D2 deiodinase as BA or fatty acids. *Ucp1* mRNA was decreased to 60% in NOD1 mice on HFD, which agrees with the observed obesity under HFD in NOD1 KO mice (Supplementary Fig. S6b). Conversely, D1 activity in liver was significantly lower in NOD1 KO animals, which correlates with the T3 levels measured in this tissue (Fig. 6d), despite to observe similar mRNA levels for *Dio1* (Supplementary Fig. S6c).

### Gut microbiota differences in WT and NOD1 KO mice under different diets

**Figure 7:**
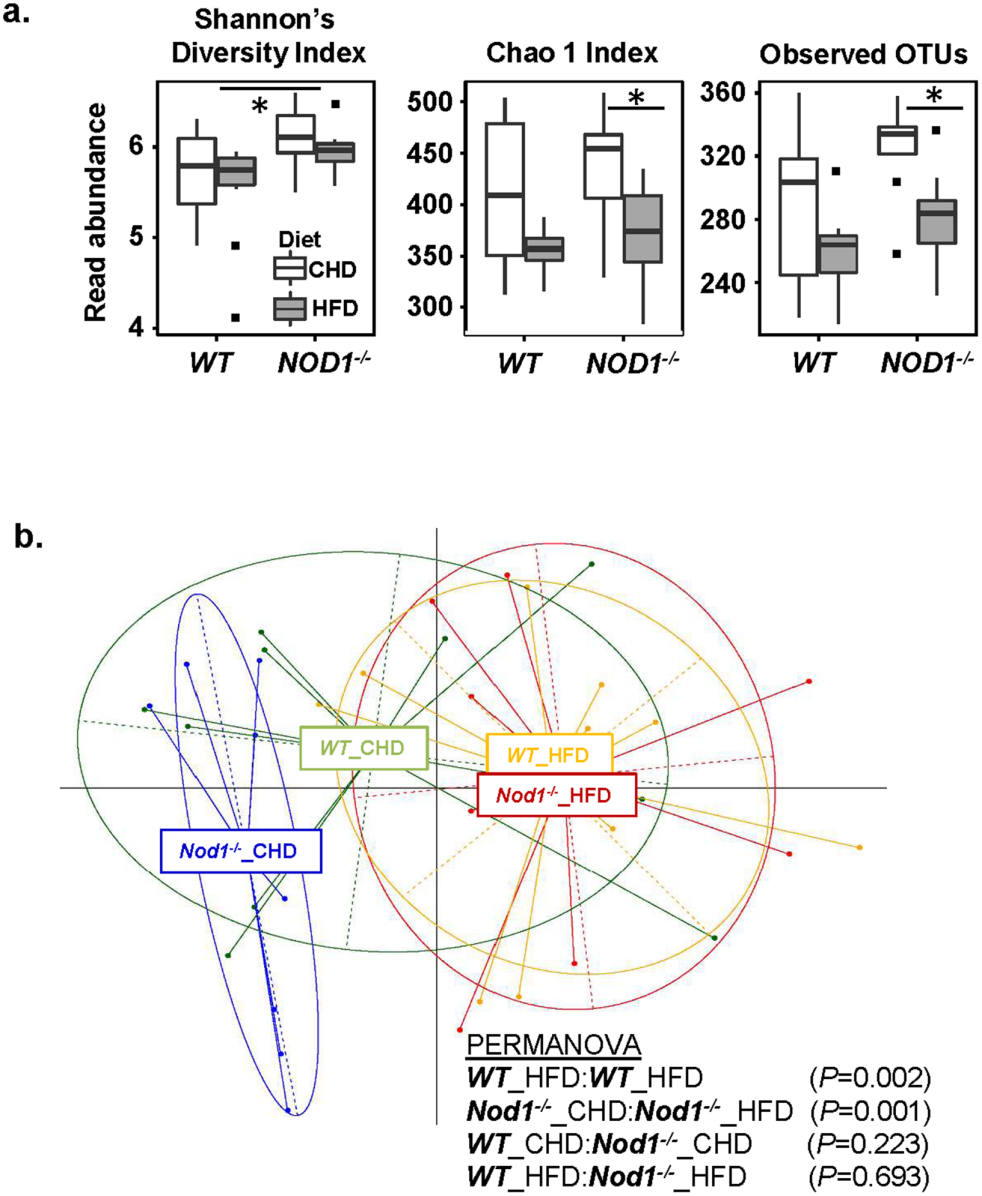
Characterization of the gut microbiota. (**a**) Alpha diversity indices (Shannon’s diversity index, Chao1, Observed OTUs) from each diet and genotype treatment group. (**b**) Principle coordinates analysis (PCoA) plot using generalized UniFrac distances comparing microbial communities from each treatment group. Group means are indicated by the center of each ellipse. Distance-based non-parametric PERMANOVA tests were conducted as pairwise comparisons of treatment groups. *P* values of all pairwise comparisons were corrected for multiple comparisons using false discovery rate.

Analyses of the intestinal microbiota were performed in the two experimental groups (NOD1 KO and WT mice) under the CHD and after switching to a HFD. These enabled us to identify differences in microbiota related to both the diet (HFD vs. CHD) and the genotype (NOD1 KO vs. WT). In general, indices of α diversity tended to be reduced by the HFD but the differences varied depending on the genotype (Fig. 7a). As shown in Fig. 7a, the HFD feeding caused decreases in observed OTUs and in the Chao1 index (a measure of species richness) in NOD1 KO mice but not in WT mice, suggesting that HFD exerted stronger adverse effects in the gut microbiota composition of the NOD1 KO. Nonetheless, Shannon’s diversity index (species diversity) was slightly lower in WT than in NOD1 KO under HFD (Fig. 7a). Comparison of β diversity showed that after a HFD, it appears that the microbiota composition changed for both genotype groups when compared to CHD. Analysis using generalized UniFrac (which down-weights abundant or rare taxonomic groups) revealed significant differences between CHD and HFD for WT and NOD1 KO groups, individually. However, no significant differences in β diversity could be attributed to the genotype, when comparing WT *vs* NOD1 KO groups for CHD and HFD, respectively (Fig. 7b). When differences in specific taxonomic categories were analyzed as a function of the genotype under each specific dietary period, it was observed that the NOD1 KO group was characterized by lower abundances of the family *S24-7*, as well as the genera *Bacteroides* and *Mucispirillum* compared to the WT group under a standard CHD (Supplementary Fig. S7). In contrast, higher abundances of the genera *Oscillospira*, *Prevotella*, [*Prevotella*], *Parabacteroides*, AF12 (family *Rikenellaceae*), *Rikenella*, *Dehalobacterium, Helicobacter*, *Desulfovibrio* and rc4_4 (family *Peptococcaceae*) were observed in the NOD1 KO group compared to the WT under a CHD. After HFD feeding, the NOD1 KO group was characterized by higher abundances of the family *Desulfovibrioaceae* and the genera *Odoribacter, Prevotella, Parabacteroides*, [*Ruminococcus*], *Rikenella, Paraprevotella* and *Helicobacter* compared to the WT group. Therefore, four bacterial groups were increased in NOD1 KO mice compared to WT regardless of the diet, including *Prevotella, Parabacteroides, Rikenella* and *Helicobacter*. The HFD feeding caused a decrease in several genera (*Bifidobacterium, Rikenella, Bilophila*, rc4_4 and *Enterococcus*) and an increase in others (*Helicobacter)* in both WT and NOD1 KO groups, compared to the groups fed the CHD, revealing the key influence of the diet on the gut microbiota composition above the genetic background. Of these bacterial groups, the initial increased levels of *Helicobacter* found in NOD1 KO mice could aggravate the effects of the HFD, which also could contribute to their increase. Specific effects of the HFD depending on the genotype were also detected. Indeed, the HFD decreased the abundance of some taxonomic genera (*Oscillospira, Coprococcus* and *Streptococcus*) only in the NOD1 KO group. Other taxonomic genera were increased only in the NOD1 KO group (*Bacteroides*, [*Ruminococcus*], *Dorea*) or the WT group (*Desulfovibrio*, *Allobaculum*) under HFD feeding. These findings suggest that the genotype influences the susceptibility of the host to diet-induced microbiota changes (Supplementary Fig. S7).

## Discussion

In this work, we show that under a standard CHD, the absence of NOD1 does not alter lipid metabolism nor promote obesity, as deduced by the presence of normal adipose tissue and complete absence of liver steatosis in these mice. However, HFD favors a rapid and significant development of obesity, or at least overweight, caused by an enlargement of eWAT adipocytes and enhanced hepatic steatosis in NOD1 KO mice, whereas this situation is delayed in the WT counterparts. Blood lipids from NOD1 KO mice fed HFD show an increase in total cholesterol, mainly due to the elevation of the HDL fraction. When animals are analyzed in metabolic cages, the absence of NOD1 results in higher REE values. However, under a HFD regimen, the rise in REE during the darkness period is lesser than in the WT animals, contributing to the increased obesity in NOD1 KO mice *vs*. the same genotype in CHD. Indeed, the significant decreased levels of *Pnpla3* in eWAT suggest a moderate hypothyroidism, as previously described^35^. In fact, among the targets responsive to thyroid hormones and involved in obesity, *Pnpla3* plays a role in the enlargement of eWAT^35^. However, the situation is more complex in view of the elevation of *Atgl* levels, a key enzyme in triglyceride lipolysis^47^, in the absence of NOD1, both under CHD and HFD *vs*. the corresponding levels in the WT counterparts.

We provide evidence of different mechanisms that could explain this accelerated obesity of NOD1 KO mice fed HFD. According to our data, one of the key players is a significant alteration of thyroid hormone homeostasis at 6 weeks after HFD. Under a CHD, NOD1 deficiency results in higher serum levels of both T3 and T4, accompanied by lower FT4 and FT3 hormones in the thyroid (hormone ready to be released). Thyroidal FT4 and FT3 are strongly decreased in NOD1 KO mice fed CHD, showing a profound effect of thyroid secretion. If similar decreases in NOD1 expression or activity were found in humans, with decreases in thyroidal FT4 and FT3, this would indicate the early involvement of the thyroid in pathological events, such as obesity associated to alterations in this NLR. This thyroid situation is reversed under HFD, which may contribute to the increased obesity of NOD1 deficient mice. The strong response of BAT D2 deiodinase points to higher T3 requirements in NOD1 KO mice. Regarding other alternatives accounting for T3 decreased levels, such as Dio3, it should be mentioned that Dio3 is expressed mainly during fetal life and in proliferative situations, including cells in culture. Dio3 is the main pathway for T3 degradation. It has been reported that Dio3 is not expressed or it is undetectable in murine BAT, thyroid and adult liver. Therefore, thyroid hormone dysfunction appears to be an early event prior to the development of insulin resistance, and related to the gain in body weight mainly through lipid accumulation in fat and liver tissues.

It is known that HFD alters the permeability of the intestinal barrier, and its integrity is compromised by this dietary regimen^24,25,54–56^. HFD lipids, in combination with bacterial molecules from the gut microbiota (bacterial cell wall components, DNA, etc.) are translocated across this barrier and gain access to systemic circulation and activate pro-inflammatory pathways, not only in immune cells but also in other tissues where NOD1 and NOD2 are expressed^11,12,50^. Our data suggest that HFD is involved in the rise of circulating NOD1 activatos as deduced by the increase in the phosphorylation of the adaptor RIPK2 in the WT mice. Interestingly, the absence of NOD1 per se has an impact on the gut microbiota composition, which exhibits a higher content in *Parabacteroides, Prevotella, Rikenella* and *Helicobacter* regardless of the diet. The differences in microbiota related to the NOD1 deletion seem to influence the subsequent HFD-induced effects on the gut ecosystem, which could contribute to aggravate the immune-metabolic phenotype of the obese mice*.* In addition to this, NOD1 deficiency in epithelial intestinal cells is likely associated with a higher translocation of gut-derived molecules that contribute to activate immunity.

Exacerbation of the adverse metabolic effects of the HFD in NOD1 KO mice compared to WT mice, including increased adiposity and lipogenesis and changes in ileum lipid composition could be related to dietary-induced alterations in the natural intestinal ecosystem of the NOD1 KO animals. This is characterized by reductions in some diversity indexes and shifts in the abundance of specific bacterial taxonomic groups. A role for dietary-induced changes in the microbiota in dietary lipid absorption and deposition in peripheral tissues has been reported in different study models^12,57^. Diet-induced increases in the phylum *Firmicutes* were shown to increased lipid absorption in the intestine through fecal transplantation experiments^57^. Other studies reported that that lipid absorption could be increased by a microbiota enriched in *Clostridiaceae* and with reduced abundance of *Bacteroidaceae* and *Bifidobacteriaceae*^12^. In our study, HFD-induced reductions in *Bifidobacterium*, which could explain increased lipid absorption leading to obesity and adiposity, although these effects were identified in both WT and NOD1 KO and attributed exclusively to the HFD. Specific dietary effects on the intestinal ecosystem of NOD1 KO mice (not detected in WT mice under HF feeding), such as reductions in *Oscillospira*, *Coprococcus* or *Streptococcus* and increases in others (*Dorea*, [*Ruminococcus*] *or Bacteroides*) could account for the stronger adverse impact of the diet on lipid absorption and metabolism than in WT mice. *Oscillospira* has often been associated with leanness in human microbiota studies^58^, although opposite associations have also been reported in WT rodents^59^. This potential benefits could be attributed to the ability of *Oscillospira* spp. to produce butyrate, which is thought to play positive roles in obesity^58^. *Oscillospira* spp. also utilize as a primary energy source host-derived glycans (glucoronate), instead of dietary starches, which will cost energy to the host and could explain its positive association with leanness in humans^58^. By contrast, increases in *Dorea* have been positively associated with obesity/adiposity in rodent^59,60^ and human^61^ studies.

Specific taxa of the gut microbiota regulate proteins involved in BA metabolism, such as BA transporters, bile salt hydrolase (BSH) and BA 7α-dehydroxylase activities. These are responsible for both BA deconjugation and subsequent dehydroxylation in the distal gut. Their activity could increase the excretion of BAs in feces and reduce their concentrations in the body, leading to induction of BA synthesis from cholesterol in the liver^11,12^. Therefore, the induction of the expression of Cyp7a1 in the liver of NOD1 KO mice under both the CHD and the HFD could be related to the specific microbiota alterations associated with the mouse genotype and the subsequent effect of the HFD. For example, *Parabacteroides *spp., which showed higher abundances in NOD1 KO mice, although were not specifically increased by the HFD, produce secondary BAs and, thereby, could contribute to inducing BA synthesis from cholesterol in the liver as well^62–64^.

Collectively, the low-grade immune response upon HFD is exacerbated in the case of NOD1 deficiency, contributing to dysfunctional lipid synthesis, due to a higher infiltration of pro-inflammatory immune cells in adipose and liver tissues^18,20^, as confirmed by the quantification of the infiltrated cells and the rise in circulating TNF-α levels. Indeed, experiments using germ-free mice show protection against HFD-induced obesity in NOD1 KO animals, although this phenotype is suppressed when mice are housed under conventional conditions and the gut becomes colonized^13,18,65–67^. The HFD used promoted a modest increase in body weight of the WT animals (9.8% increase after 6 weeks); however, in the absence of NOD1 the body weight gain was 33.3% during this period, reflecting this obesity in adipose tissue and in the liver. In addition to this, these tissues became more infiltrated by immune cells, such as macrophages and neutrophils, as it has been previously described^4,22,24^. The idea that lipids and bacterial-derived molecules cooperate in the induction of ‘metabolic inflammation’ and obesity comes from the fact that particularly NOD1 is overexpressed and activated in adipose tissue in response to these challenges^10,18,20,23^. However, our data provide a different scenario since constitutive deficiency of NOD1 worsens the metabolic dysfunction due to HFD. In fact, NOD1 polymorphisms in humans -associated with differences in the activity of this NLR- appear to play a relevant role in inflammatory-related diseases, including obesity^23,68,69^.

Regarding insulin resistance and NLR activity, other groups have described that NOD1 activation after iE-DAP administration promotes insulin resistance^20,23,24,70^. However, in animals lacking NOD1 in the hematopoietic system, Chan et al.^18^ reported that after 18 weeks of HFD, the WT animals exhibited hyperinsulinemia, insulin resistance and a reduced capacity to decrease glucose overload, a situation that was significantly more benign in the hematopoietic NOD1 deficient mice. Here we provide a new piece of knowledge since our animals deficient in NOD1 and fed a HFD for 6 weeks, despite an evident obesity, are not resistant to insulin and exhibit an improved glucose tolerance test when compared with the WT counterparts. These results suggest that additional dysfunctional changes are required to establish insulin resistance and defective oral glucose tolerance test, compatible with diabetes progression in WT animals. Moreover, reversion to CHD after 6 weeks of HFD shows a significant recovery in the liver steatosis of the NOD1 KO animals and almost complete absence of Oil-red O staining in the WT counterparts, which is in agreement with the failure of the establishment of the insulin-resistance phenotype in these 6 weeks of HFD regimen.

Finally, although less attention has been paid to thyroid metabolism associated with microbial composition it remains a relevant pathophysiological issue as observed from our results in the NOD1 KO mice, and discussed in a recent review^71^. Indeed, our data suggest an interplay between NOD1 activity, microbiota and thyroid hormone function that might help to unravel unexpected obesity-related pathologies in humans.

## Supplementary information

Supplementary Information.

## Abbreviations

HFD: High fat diets
JNK: C-Jun N-terminal kinase
KO: Knockout
MAPK: Mitogen-activated protein kinase
q(RT)-PCR: Quantitative real-time (RT) polymerase chain reaction
SDS-PAGE: Sodium dodecyl sulfate polyacrylamide gel electrophoresis
WT: Wild-type
