# Supplementary material for: NOD1 deficiency promotes an imbalance of thyroid hormones and microbiota homeostasis in mice fed high fat diet
Source: Sci Rep. 2020 Jul 23;10:12317. doi: 10.1038/s41598-020-69295-2 (PMC7378078; doi:10.1038/s41598-020-69295-2)

NOD1 deficiency promotes an imbalance of thyroid hormones and microbiota homeostasis in mice fed high fat diet

[1,2]Silvia González-Ramos[#]*,[1]Marta Paz-García*, [1]Victoria Fernández-García*, [3]Kevin J. Portune, [4]Emilio F. Acosta-Medina, [3]Yolanda Sanz, [1,5]Antonio Castrillo, [1,2,5]Paloma Martín-Sanz, [1]Maria Jesus Obregon, and [1,2,5]Lisardo Boscá[#]

[1]Instituto de Investigaciones Biomédicas Alberto Sols (CSIC-UAM), Arturo Duperier 4, 28029 Madrid, Spain.
[2]Centro de Investigación Biomédica en Red de Enfermedades Cardiovasculares (CIBERCV), y Hepáticas y Digestivas (CIBEREHD), ISCIII; Spain.
[3]Microbial Ecology, Nutrition & Health Research Unit, Institute of Agrochemistry and Food Technology, National Research Council (IATA-CSIC), Valencia, Spain.
[4]Center for Genetic Engineering and Biotechnology, Havana, Cuba.
[5]Unidad Asociada de Biomedicina. Instituto de Investigaciones Biomédicas Alberto Sols (CSIC-UAM) and Universidad de Las Palmas, Gran Canaria, Spain

Supporting Information

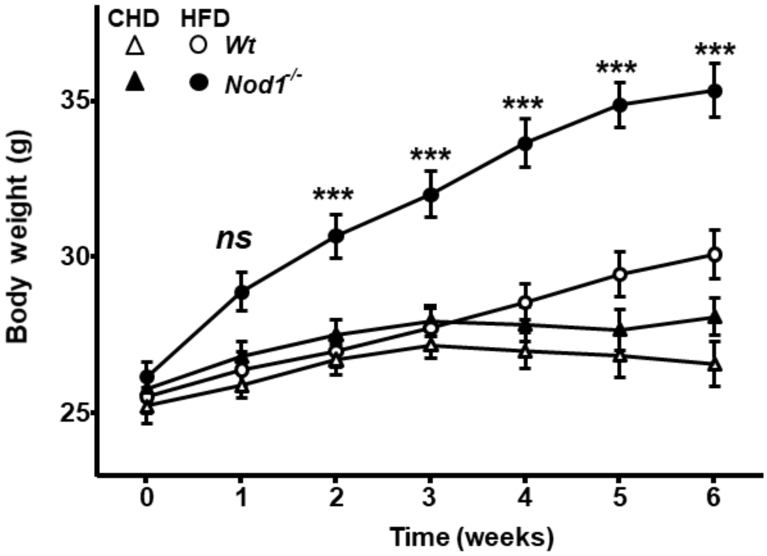

b.

Tissue (mg)	CHD		HFD	
	Wt	*Nod1*$^{-/-}$	*Wt*	*Nod1*$^{-/-}$
BAT	118 ± 10	134 ± 13	139 ± 5	173 ± 6 ***
eWAT	420 ± 50	602 ± 93	544 ± 52	1165 ± 114 ***
iWAT	310 ± 34	394 ± 39	434 ± 44	919 ± 137 *
Liver	1266 ± 49	1100 ± 29	1477 ± 50	1752 ± 89
Tibia	21.7 ± 0.2	21.5 ± 0.2	21.7 ± 0.1	21.1 ± 0.1

Figure S1. Changes in body weight of WT and NOD1 KO mice under CHD or fed HFD. The data show the weights of 15 mice per condition (**a.**) and the tissue weight at week 6 of follow-up, expressed as mg of tissue per animal (**b.**). Results show the mean±S.E.M. per nutritional condition and genetic background. *$P<0.05$; ****$P<0.005$ *vs*. the corresponding condition in WT mice. ***ns***, not statistically significant.

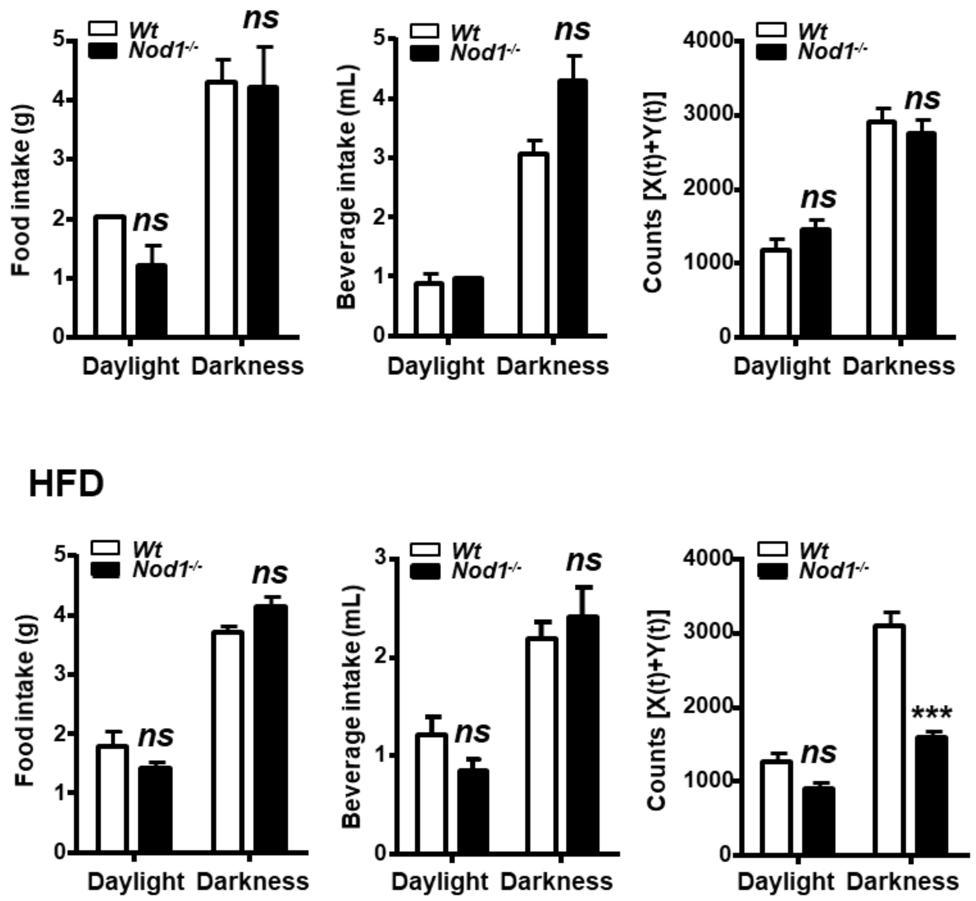

Figure S2. Evaluation of animal parameters after CHD or HFD in metabolic cages. Animals were fed under the indicated regimes and the food and beverage intake, and the movement were recorded in periods of 12h. Results show the mean±S.E.M. of 6 animals per nutritional condition and genetic background. ****P<0.005 vs*. the corresponding condition in WT mice. ***ns***, not statistically significant.

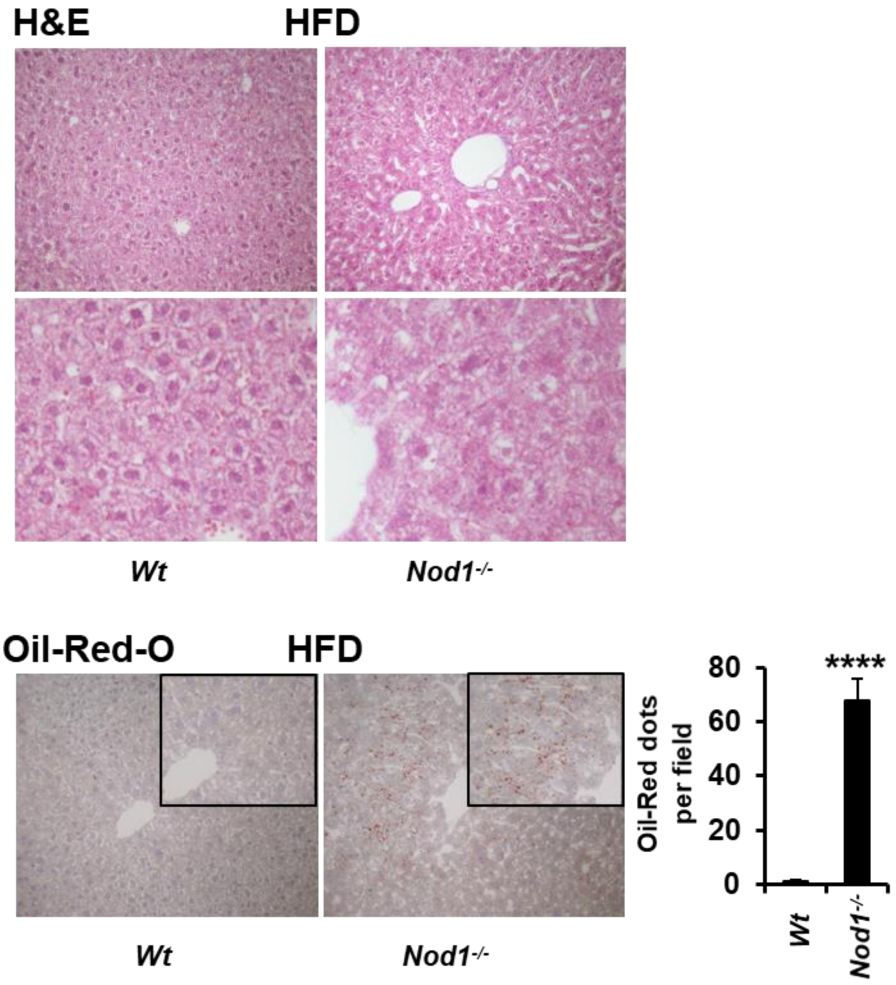

Figure S3. Recovery of WT and NOD1 KO mice after HFD. Animals (n=4 for each condition) were fed for 4 weeks HFD. After feeding for two additional weeks with CHD, samples of liver were analyzed by eosin and hematoxylin staining or with Oil-red O staining to evaluate lipid accumulation. The number of oil red ‘dots’ per field was quantified. Results show a representative histochemistry and the mean±S.E.M. of the red dots in at least four equivalent fields. ****$P<0.001$ *vs*. the corresponding condition in the dietary reversed WT mice.

Figure S4.

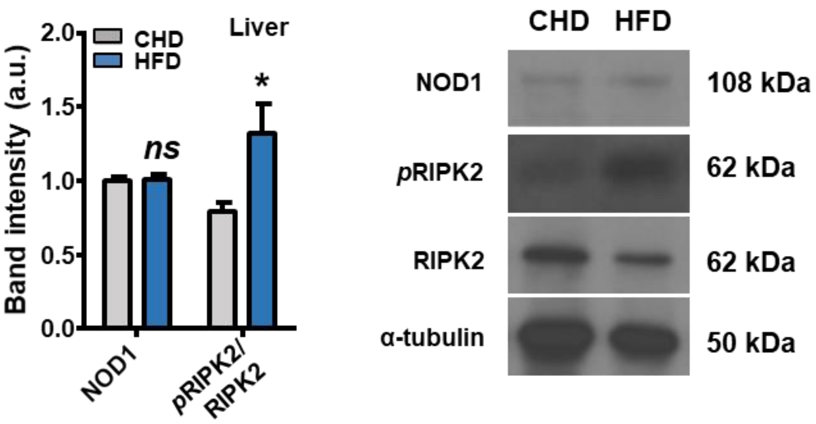

Figure S4. HFD activates NOD1 in WT mice. Animals were fed HFD for 6 weeks and the levels of liver NOD1, phopho-RIPK2 and RIPK2 were determined by immunoblot. Results show a representative blot and the mean±S.E.M. from 6 animals. *$P<0.05$ *vs*. the corresponding condition in CHD. ***ns***, not statistically significant.

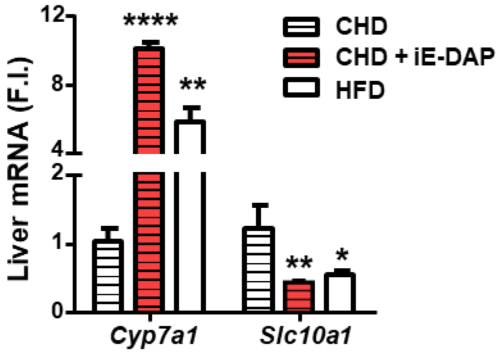

Figure S5. NOD1 activation under CHD promotes changes in hepatic bile acid metabolism markers similar to those achieved under HFD. Animals were fed CHD and administered for 24h the NOD1 activator iE-DAP, or fed HFD for 6 weeks. The hepatic mRNA levels of *Cyp7a1* and *Slc10a1* were determined. Results show the mean±S.E.M. of 6 animals. *$P<0.05$, **$P<0.01$, ****$P<0.001$ *vs*. the CHD condition.

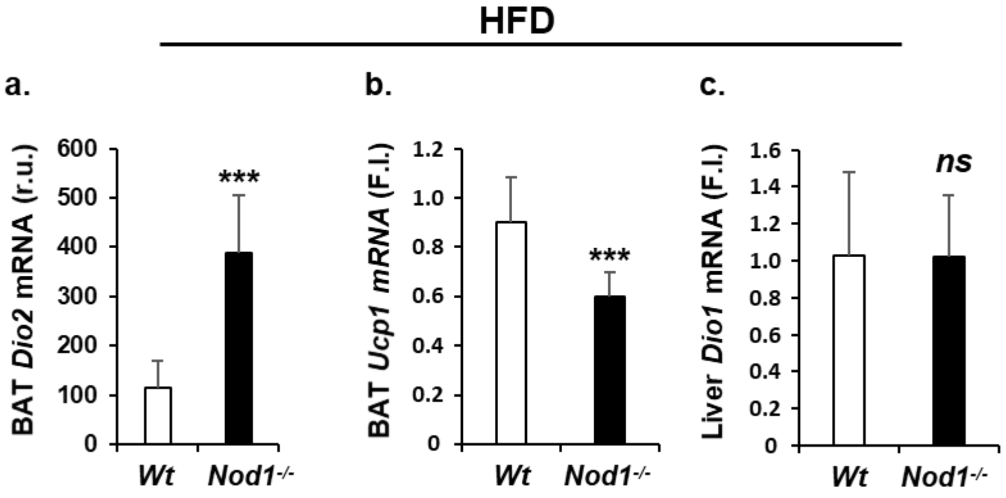

Figure S6. mRNA levels of *Ucp1* and *Dio2* in BAT and *Dio1* in liver in WT and NOD1 deficient mice under HFD. Animals were fed HFD for 6 weeks and the levels of the corresponding genes were determined using specific Taqman probes (**a.-c.**). Results show the mean±S.E.M. of 6 animals. ****P<0.005 vs*. the WT condition. ***ns***, not statistically significant.

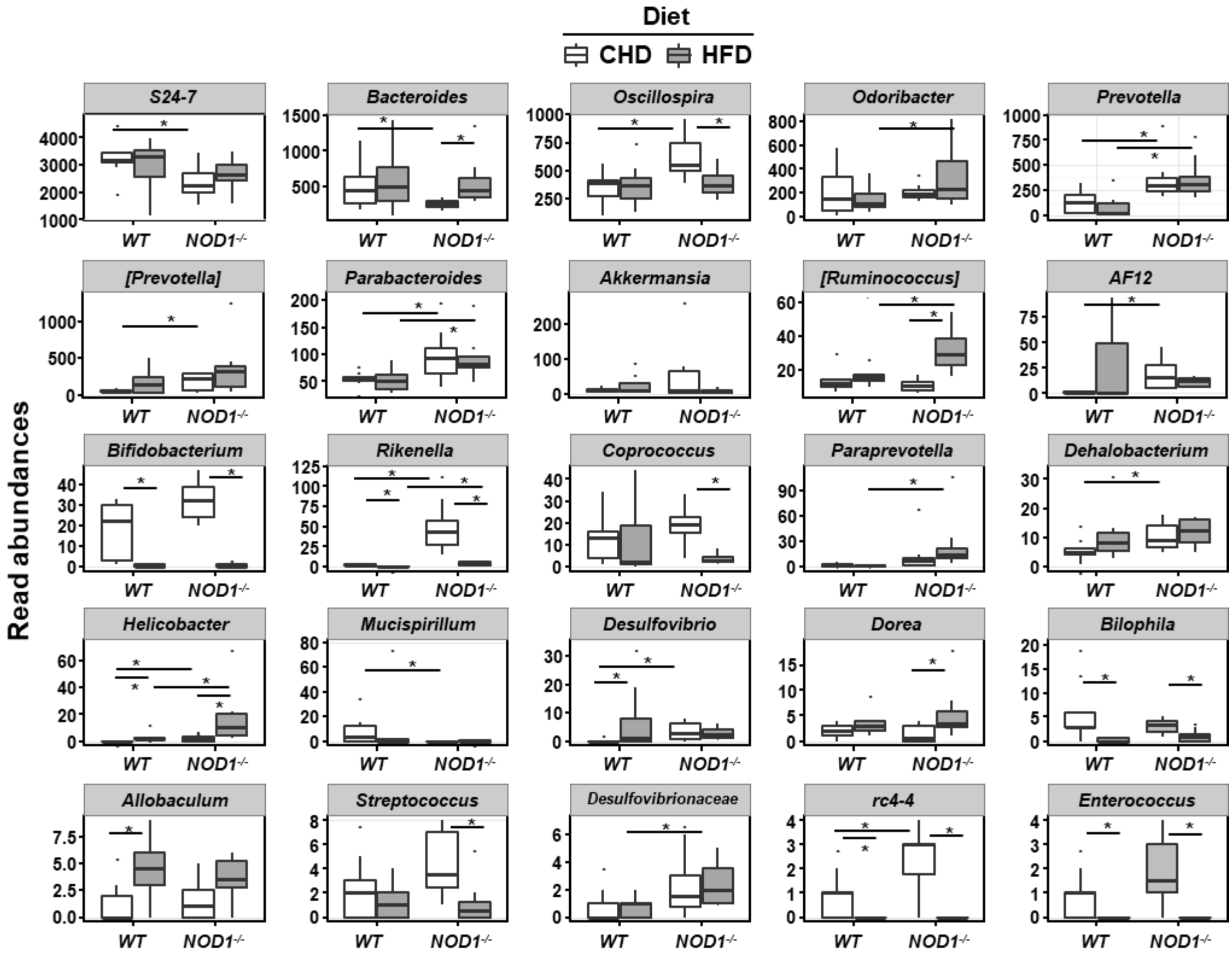

Figure S7. Microbiota taxonomic abundances. Boxplots of gut microbiota taxonomic groups (family and genera) in which at least one significant difference was detected between treatment groups. Significant differences (*P<0.05*) between respective treatment groups are indicated with a bar and an asterisk (*).

Figure 3c

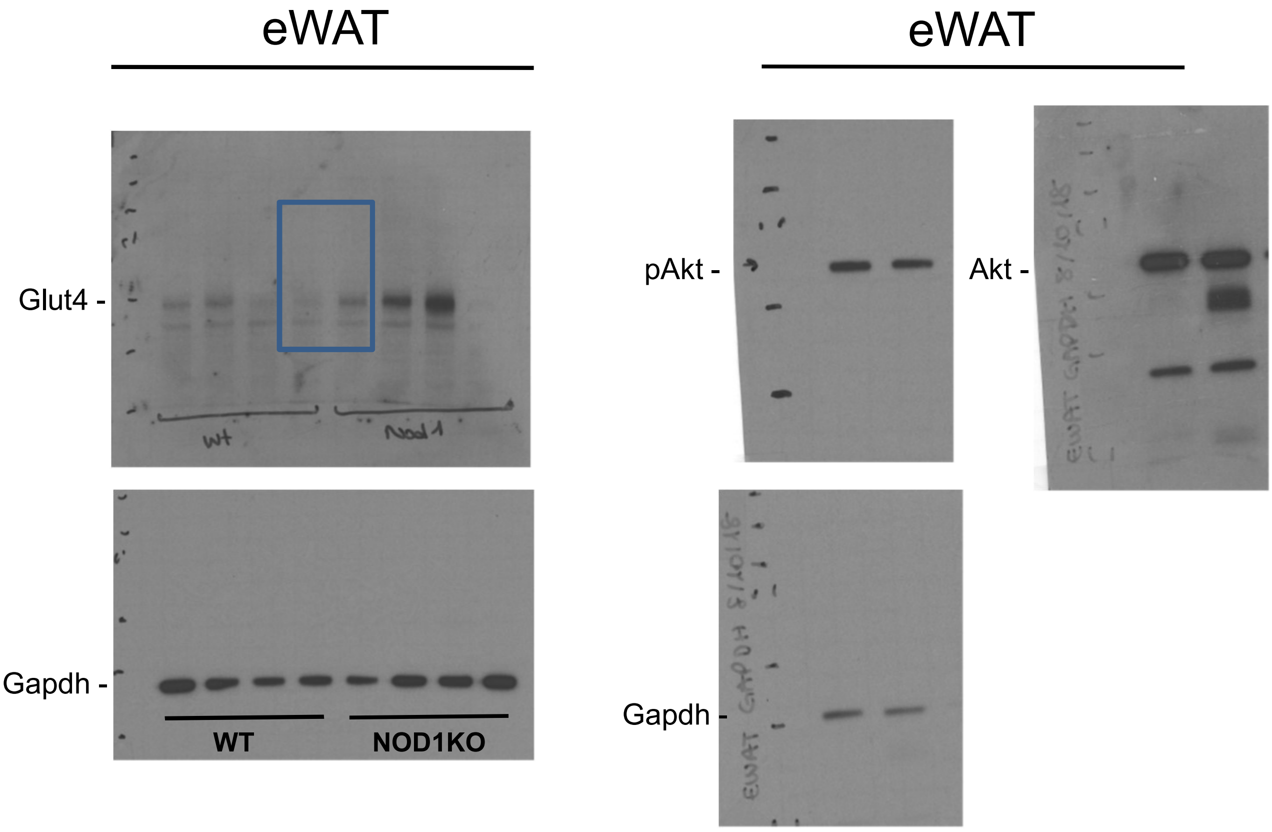

Figure 4a

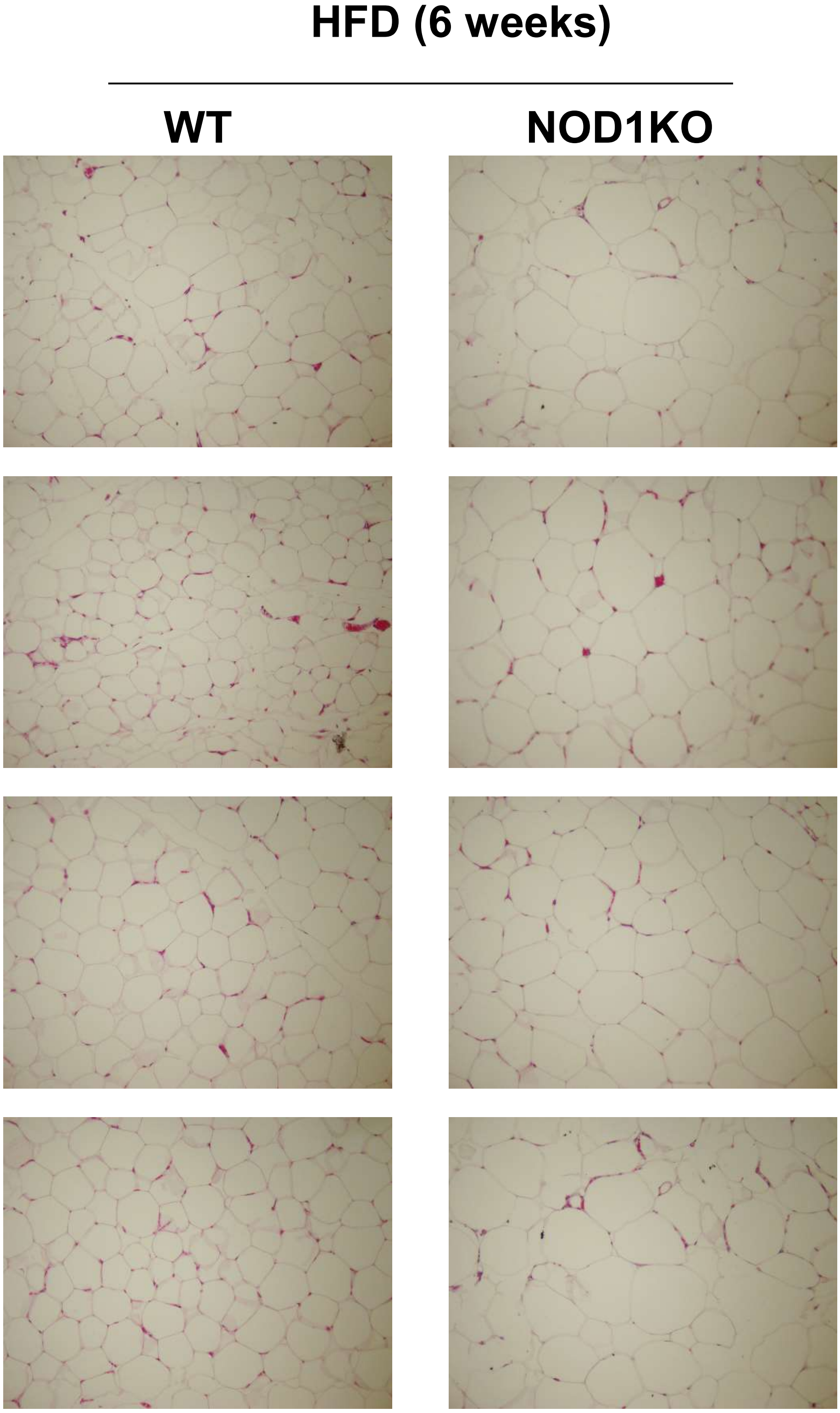

Figure 4d

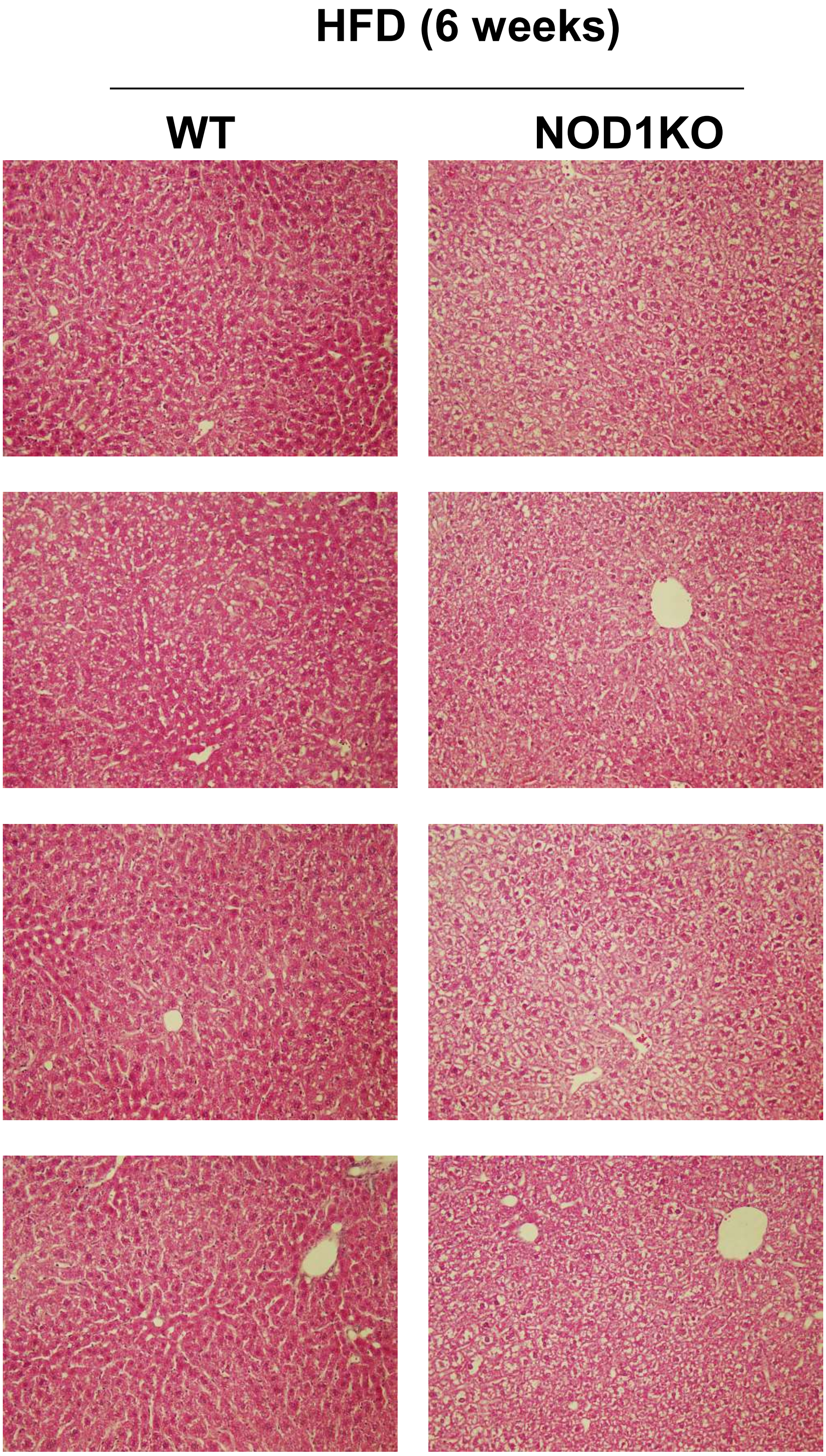

Suppl. Figure S3

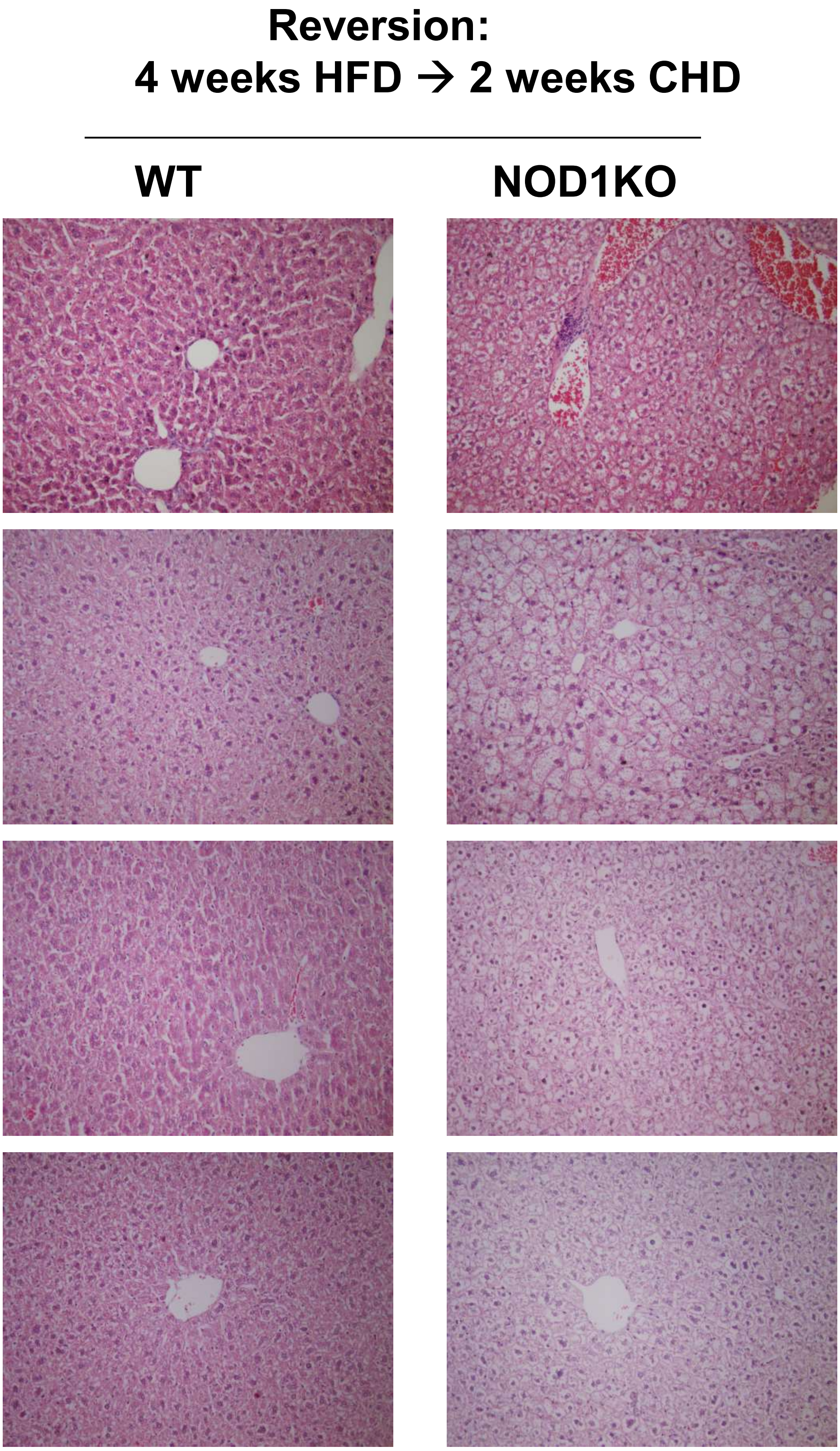

Suppl. Figure S4

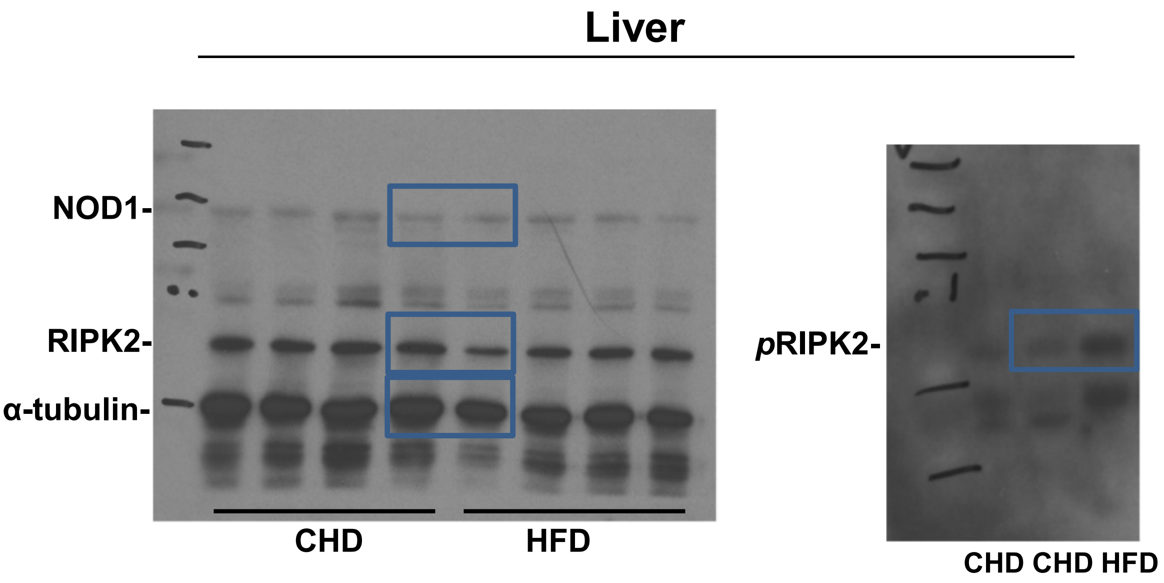

Supplement: Supplementary file 1 — Supplementary Information. [file 41598_2020_69295_MOESM1_ESM.pdf]
